# Mussel-Inspired Catechol Functionalisation as a Strategy to Enhance Biomaterial Adhesion: A Systematic Review

**DOI:** 10.3390/polym13193317

**Published:** 2021-09-28

**Authors:** Pedro M. Costa, David A. Learmonth, David B. Gomes, Mafalda P. Cautela, Ana C. N. Oliveira, Renato Andrade, João Espregueira-Mendes, Tiago R. Veloso, Cristiana B. Cunha, Rui A. Sousa

**Affiliations:** 1 Stemmatters, Biotecnologia e Medicina Regenerativa SA, Parque de Ciência e Tecnologia Avepark, Zona Industrial da Gandra, 4805-017 Barco, Portugal; david.learmonth@iplantprotect.pt (D.A.L.); dbgomes@stemmatters.com (D.B.G.); mpcautela@stemmatters.com (M.P.C.); acoliveira@stemmatters.com (A.C.N.O.); rafaba2@gmail.com (T.R.V.); cbcunha@stemmatters.com (C.B.C.); rasousa@stemmatters.com (R.A.S.); 2 Clínica do Dragão, Espregueira-Mendes Sports Centre-FIFA Medical Centre of Excellence, 4350-415 Porto, Portugal; randrade@espregueira.com (R.A.); jem@espregueira.com (J.E.-M.); 3 Dom Henrique Research Centre, 4350-415 Porto, Portugal; 4 Faculty of Sports, University of Porto, 4200-450 Porto, Portugal; 5 ICVS/3B’s-PT Government Associate Laboratory, Braga, Portugal; 6 Life and Health Sciences Research Institute (ICVS), School of Medicine, University of Minho, 4710-057 Braga, Portugal

**Keywords:** biomaterial, dopamine, catechol, adhesion, functionalisation

## Abstract

Biomaterials have long been explored in regenerative medicine strategies for the repair or replacement of damaged organs and tissues, due to their biocompatibility, versatile physicochemical properties and tuneable mechanical cues capable of matching those of native tissues. However, poor adhesion under wet conditions (such as those found in tissues) has thus far limited their wider application. Indeed, despite its favourable physicochemical properties, facile gelation and biocompatibility, gellan gum (GG)-based hydrogels lack the tissue adhesiveness required for effective clinical use. Aiming at assessing whether substitution of GG by dopamine (DA) could be a suitable approach to overcome this problem, database searches were conducted on PubMed^®^ and Embase^®^ up to 2 March 2021, for studies using biomaterials covalently modified with a catechol-containing substituent conferring improved adhesion properties. In this regard, a total of 47 reports (out of 700 manuscripts, ~6.7%) were found to comply with the search/selection criteria, the majority of which (34/47, ~72%) were describing the modification of natural polymers, such as chitosan (11/47, ~23%) and hyaluronic acid (6/47, ~13%); conjugation of dopamine (as catechol “donor”) via carbodiimide coupling chemistry was also predominant. Importantly, modification with DA did not impact the biocompatibility and mechanical properties of the biomaterials and resulting hydrogels. Overall, there is ample evidence in the literature that the bioinspired substitution of polymers of natural and synthetic origin by DA or other catechol moieties greatly improves adhesion to biological tissues (and other inorganic surfaces).

## 1. Introduction

The concept of regenerative medicine has been around for many decades. From the capacity of newts (i.e., small urodele amphibians) to repeatedly regenerate their limbs [1], to human organ and bone marrow transplantation procedures [2], there is abundant evidence in nature and clinical practice of the ability to regenerate dysfunctional or diseased organs and tissues. The success of more advanced regenerative medicine approaches, including tissue and organ engineering, is nevertheless highly dependent on the design of systems containing cells, biomaterials and biological clues that are capable of replicating the target tissues and surrounding milieu, attracting endogenous multipotent stem cells and stimulating growth and repair. Versatile biomaterials with adequate mechanical and physicochemical properties, capable of supporting cell survival, proliferation and encouraging autologous tissue growth are therefore essential for the development of safe and effective regenerative medicine-based therapeutic solutions.

**Figure 1:**
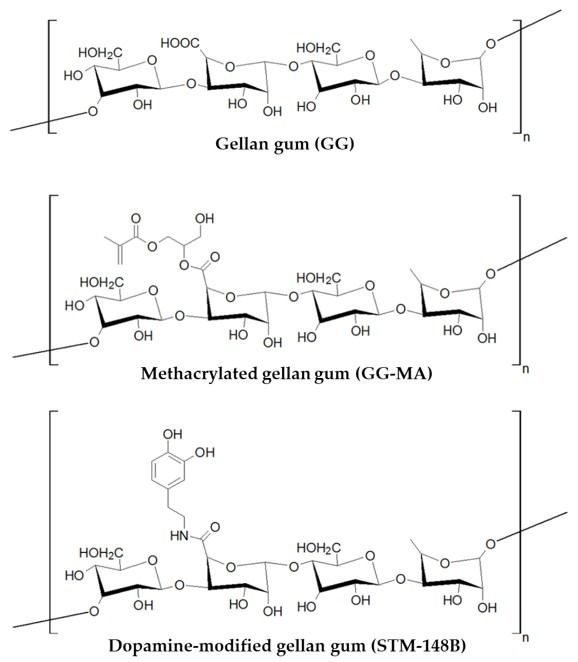
Chemical structure of the tetrasaccharide repeat unit of GG, its semi-synthetic derivative GG-MA and dopamine-modified GG (STM-148B).

The interaction of biomaterials with surrounding structures is dependent on a wide range of features, including size, surface charge, hydrophobicity, and water content, among others [3]. For this reason, the design, architecture and surface modification of biomaterials are critical to ensure perfect integration with living systems [4]. Over the last decade, tremendous advances have been achieved in the development of biomaterial-based constructs, including hydrogels, scaffolds, foams, films and composites, using polymers derived from natural or synthetic sources. Among the naturally sourced polymers described in the literature for biomedical applications, gellan gum (GG, Figure 1), a Sphingomonas elodea-derived anionic polysaccharide, with a linear structure consisting of repeats of the tetra-saccharide “D-glucose–D-glucuronic acid–D-glucose–L-rhamnose”, has been presented as a promising biomaterial [5,6]. Indeed, GG presents interesting physicochemical properties, biocompatibility and a versatile backbone that includes multiple reactive hydroxyl and carboxyl groups per unit [6]. Nevertheless, unmodified GG is severely limited by (i) low aqueous solubility (requiring heating to around 90 °C for preparation of solutions at 1%), (ii) inconvenient thermo-reversible gelation above physiological temperature (40–42 °C), and (iii) limited adhesiveness to biological structures. For this reason, GG has been modified to optimise its physicochemical and biological properties. The semi-synthetic methacrylated GG (GG-MA, Figure 1), obtainable by the reaction of GG with glycidyl methacrylate [7], displays enhanced water solubility (so that solutions at 1–2% can be prepared in water at room temperature) and is capable of crosslinking mediated by monovalent and divalent cations, as well as by ultraviolet light (due to the methacrylate substituent). Both ionic- and photo-crosslinked GG-MA hydrogels have been shown to be biocompatible [8], with improved in vivo performance reported for cartilage repair procedures [9]. Despite improvements in terms of the aqueous solubility and physicochemical properties of GG and its second-generation derivative GG-MA, the tissue adhesiveness of native and semi-synthetic GG hydrogels remains problematic, especially for applications that require integration of the hydrogels with surrounding structures, such as cartilage.

Building on the impressive adhesive capacity of certain organisms in a wet environment, the development of more surface-adherent biomaterials has focused on biomimetic approaches. Particular focus has been paid to the molecular mechanisms mediating the adhesion of marine mussels to slippery surfaces. Their remarkable capacity to establish stable long-term attachment has been attributed to the production of adhesive proteins in adhesive pads, containing high levels of the catecholamine-rich amino acid 3,4-dihydroxy-L-phenylalanine (L-DOPA). Catecholamine is responsible for mediating adhesion through a mechanism involving the coordination of metal cations present in seawater leading to crosslinking and the formation of a sticky, water-resistant glue [10,11].

The premise of mimicking natural adhesion mechanisms has been explored with mussel-inspired semi-synthetic polymers for biomedical applications to improve the adhesive properties of hydrogels. The wealth of literature evidence for the augmentation of biopolymer adhesiveness to biological tissues through catechol modifications of polymers of natural and synthetic origin has been systematically reviewed and evaluated, and is summarised herein. Database mining activities were performed in PubMed^®^ and Embase^®^ databases, with subsequent data processing and manuscript analysis for selection of those complying with the search parameters and scope of activities. Importantly, the findings of this systematic literature supported the rationale for the synthesis and development of STM-148B (Figure 1) as a DA-modified, third-generation GG derivative with putative improved adhesive properties compared to native GG and later predecessors.

## 2. Materials and Methods

The systematic literature review was conducted in accordance with the Preferred Reporting Items for Systematic Reviews and Meta-Analyses (PRISMA) statement [12].

### 2.1. Search Strategy

The systematic literature search was performed in PubMed^®^ and Embase^®^ from database inception to 2 March 2021. Manuscripts not indexed to these databases or published after 2 March 2021 were not considered. The search terms and keywords were fine-tuned to explicitly infer that the substituent conferring improved adhesion properties must be covalently bound or attached to the polymer system, and included: dopamine, catechol, biomaterial, biopolymer, scaffold, matrix, hydrogel, device, modified, conjugated, attached, functionalized, covalent, biomimetic, mussel, bioinspired and adhesive.

The final search terms adopted (listed below) yielded an unedited total of 398 manuscripts in PubMed^®^ and 300 in Embase^®^.

(dopa*[Title/Abstract] OR catechol[Title/Abstract]) AND (biomaterial OR biopolymer OR scaffold OR matrix OR hydrogel OR device)) AND (modified OR conjugated OR attached OR functionalized OR covalent)) AND (biomimetic OR mussel OR bioinspired OR adhes*)

The detailed search strategy can be found in Table S1.

### 2.2. Selection Process

All records were exported to Mendeley Reference Manager (Elsevier^®^), and duplicates were checked and removed manually. To minimise the risk of bias, all titles and abstracts were independently screened for inclusion by two independent reviewers (P.M.C and D.A.L., D.B.G., M.P.C. or A.C.N.O.), with any discording assessments resolved by a third independent reviewer (D.B.G., M.P.C. or A.C.N.O). The full texts of all potentially relevant studies were obtained and screened for eligibility by the same reviewing team. The inclusion criteria were: (1) English language studies, and (2) those published as full manuscripts in peer-reviewed journals. The exclusion criteria were: (1) pharmaceuticals, (2) excipients, (3) active ingredients, (4) polydopamine, (5) coatings, and (6) review articles. Moreover, studies for which a full-text version was not available (e.g., conference abstracts), as well as those outside the scope of this literature review, including (1) dopamine detection or biosensing, (2) dopants (i.e., impurity elements introduced into a chemical material to alter its electrical/optical properties), (3) fouling (i.e., the accumulation of unwanted residues in solid surfaces) and anti-fouling systems, and (4) mechanistic studies (i.e., those related with the neurotransmissor dopamine) were also excluded.

Reference lists from review manuscripts (excluded at this stage) were also manually screened for additional potentially relevant studies, with two reports included for further analysis (thus increasing the total number of screened manuscripts to 700).

### 2.3. Data Extraction and Analysis

Two independent reviewers (P.M.C. and D.A.L., D.B.G., M.P.C. or A.C.N.O.) extracted all data from the manuscripts that complied with the inclusion criteria to an Excel file, identifying pre-determined items of interest, namely, (1) target application, (2) type of polymer, (3) catechol substituent, (4) type of chemistry used for conjugation of the catechol substituent, (5) crosslinking mechanism (i.e., ionic, oxidative, enzymatic or other), (6) adhesion tests and strength values, and (7) important study findings (e.g., mechanical properties, swelling, degradability, biocompatibility, etc.). Selected references with relevant usable data to be included in the literature review were then individually evaluated by two independent reviewers (to minimise the risk of bias) and analysed in terms of their scientific relevance and rigor, the validity of conclusions drawn by the authors, and the identification of any limitations or potential errors in the data, as described in Section 2.4.

### 2.4. Methodological Quality

Two independent reviewers (P.M.C. and D.A.L., D.B.G., A.C.N.O. or M.PC.) assessed the methodological quality of all included articles, in accordance with the criteria listed in Table 1. Discourse, where applicable, was resolved by a third reviewer (D.B.G., A.C.N.O. or M.P.C.).

The appraisal process for manuscript assessment (Table 1) generated a total score within the range of 4 to 8 for each article (1 for “Yes” and 2 for “No”). The lower the total score, the higher the relevance of the data for manuscript inclusion (Table 2). All references classified with “High relevance” or “Relevant” were selected for final inclusion in this systematic review, whereas references classified with “Low relevance” or “Negligible relevance” were excluded.

## 3. Results

**Figure 2:**
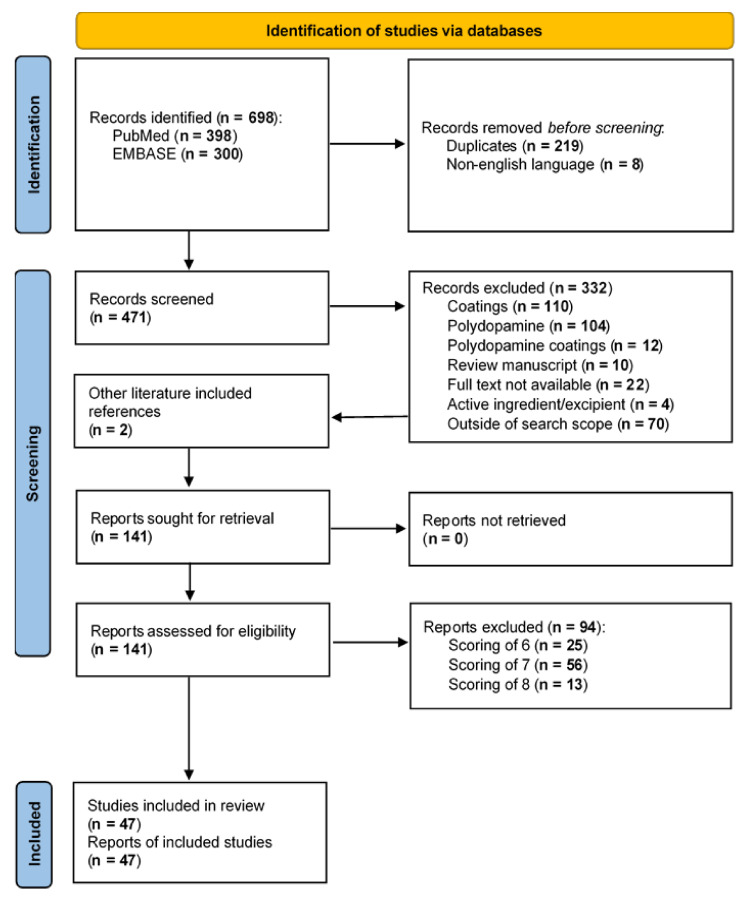
PRISMA flow chart. Adapted from [12].

**Figure 3:**
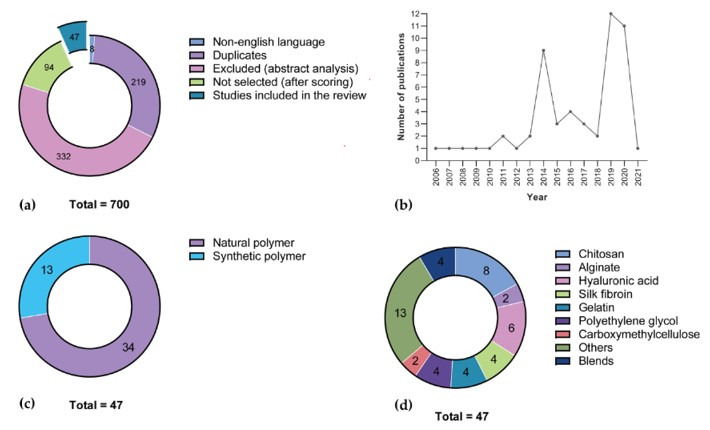
Graphical illustration of the screening process and selected manuscripts. (**a**) General overview of the screening process, including the number of manuscripts removed during identification (i.e., duplicates and those not written in the English language), abstract screening and scoring. Graphical illustration of the final group of selected manuscripts per (**b**) year of publication, (**c**) polymer source (natural vs. synthetic), and (**d**) type of polymer.

The database search yielded a total of 698 titles and abstracts (with a further 2 identified during manual search of the reference list of excluded review manuscripts). After removing duplicates and manuscripts not written in the English language, 471 abstracts were screened, and 141 full-text articles were reviewed for inclusion. Of those, 47 studies (~7% of total search results) met the eligibility criteria and were included in this review (Figure 2 and Figure 3a).

**Figure 4:**
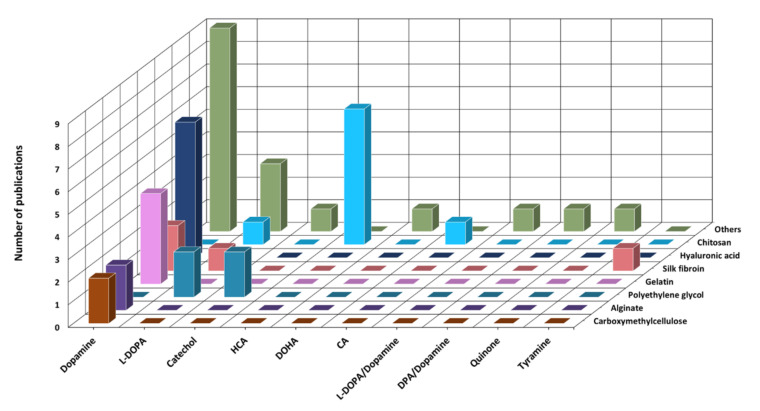
Graphical illustration of the final group of selected manuscripts based on the type of substituent per polymer. L-DOPA: 3,4-dihydroxy-L-phenylalanine; HCA: hydrocaffeic acid; DOHA: dihydroxybenzenepropionic acid; CA: caffeic acid; DPA: 3,4-dihydroxyphenyl propionic acid. L-DOPA/Dopamine; and DPA/Dopamine: modification with both substituents.

Among the manuscripts selected for inclusion, a large majority corresponded to studies dated from 2014, 2019 and 2020 (32/47, ~68%; Figure 3b) using naturally sourced polymers (34/47, ~72%; Figure 3c), and predominantly the shellfish-derived polysaccharide chitosan, either per se (8/47) or blended with other polymers (3/47, for a total of 11/47, ~23% of total, ~32% of natural polymers; Figure 3d). Moreover, chitosan and silk fibroin were found to be the most versatile polymers, accounting for modification with three different catechol substituents (Figure 4).

**Figure 5:**
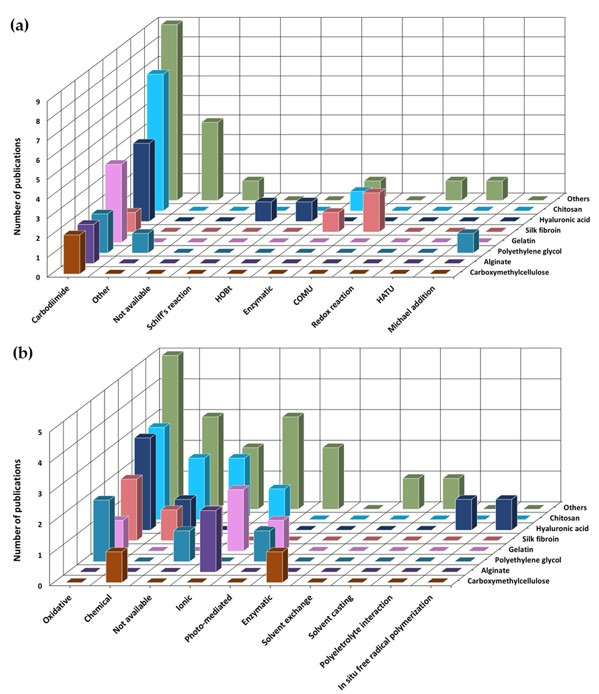
Graphical illustrations of the selected manuscripts. Graphical illustration of the final group of selected manuscripts per (**a**) coupling chemistry for catechol conjugation and (**b**) crosslinking mechanism. HOBt: N-hydroxybenzotriazole; COMU: 1-Cyano-2-ethoxy-2-oxoethylidenaminooxy)dimethylamino-morpholino-carbenium hexafluorophosphate; HATU: 1-[Bis(dimethylamino)methylene]-1H-1,2,3-triazolo[4,5-b]pyridinium 3-oxide hexafluorophosphate.

Modification of the polymer backbone with catechol moieties was predominantly performed by conjugations of dopamine (27/47, ~57%) or L-DOPA(7/47, ~15%; Figure 4) via carbodiimide chemistry (31/47, ~66%; Figure 5a), with gelation induced mostly by oxidation (16/47, ~34%; Figure 5b).

**Figure 6:**
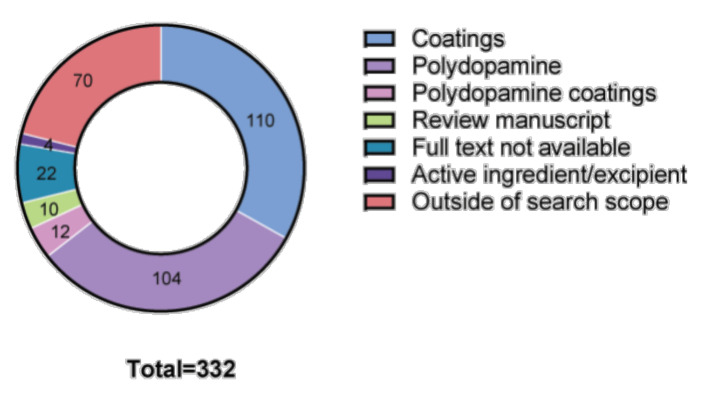
Graphical illustration of the manuscripts excluded during initial abstract assessment.

Among the manuscripts excluded during initial assessment, a significant majority involved the development of material coatings (110/332, ~33%; Figure 6) or use of polydopamine (104/332, ~31%; Figure 6).

### 3.1. Manuscripts Selected for Inclusion

A brief summary of the main findings for each selected manuscript is presented in Table 3 (naturally sourced polymers) and Table 4 (synthetic polymers), with descriptive findings included below.

#### 3.1.1. Natural Origin Polymers

Sourced from animal, plant or bacterial sources, natural polymers display favourable physical and chemical properties for applications in regenerative medicine. In the context of this literature review, two main groups of natural polymers were predominant in the final list of references: (i) polysaccharides, including chitosan, hyaluronic acid, alginate and carboxymethylcellulose; and (ii) proteins, namely, gelatine and silk fibroin. Target areas for application included tissue regeneration, wound healing and bioadhesives.

##### Chitosan (Chi)

Catechol modification of Chi, a linear polysaccharide obtained from the outer skeleton of shellfish and composed of randomly distributed β-linked D-glucosamine and N-acetyl-D-glucosamine monomers, has been extensively tested for the development of bioadhesives with potential applications in wound healing and tissue regeneration. 

Aiming at developing a new generation of tissue adhesives that overcome the drawbacks of the existing clinical options (i.e., fibrin sealants and cyanoacrylates), Zeng et al. [17] synthesised a thiol-grafted catechol-conjugated Chi derivative (ChiDS) via standard EDC chemistry (reaction with dihydrocaffeic acid and acetyl-L-cysteine for catechol and thiol addition, respectively), with the contents of thiol (215 µmol/g) and catechol groups (75 µmol/g) determined by UV–Vis spectroscopy. Gelation of ChiDS hydrogels was promoted by sodium periodate (NaIO_4_) in phosphate-buffered saline (PBS) solution, with results showing that ChiDS solutions at 4% (*w/V*) could rapidly form hydrogels (within 1 min of mixing), with the modulation of gelation times achieved by altering the ChiDS concentration and molar ratio of periodate/catechol. Similarly, rheological studies showed that the mechanical properties of hydrogels depend on the concentration of ChiDS and the molar ratio of periodate/catechol, with more catechol groups and higher molar ratios resulting in hydrogels with stronger crosslinking density. Adhesion (lap shear) strength tests were performed in porcine skin, to mimic human tissue, with adhesives showing strengths as high as 50 kPa, which were proportional to the concentration of ChiDS. In vitro assays did not show significant toxicity towards L-929 mouse fibroblast cells, thus suggesting that these hydrogels present optimal functional properties, including fast curing times, mechanical strength, high adhesion and biocompatibility.

Similarly, Ryu et al. [16] designed injectable and thermosensitive hydrogels, to be used as tissue adhesives and haemostatic materials, by combining Chi with Pluronic (a synthetic polymer composed of triblocks of polyethylene oxide-polypropilene oxide-polyethylene oxide, PEO-PPO-PEO). For this purpose, Chi was functionalised with catechol groups by EDC/NHS-mediated reactions with hydrocaffeic acid (HCA) (DS = 9.9–14.8%), and further crosslinked with terminally thiolated Pluronic F-127 triblock copolymer (PluSH), to produce temperature-sensitive and adhesive sol–gel transition hydrogels. Indeed, although catechol-conjugated Chi (Chi-C) was capable of gelation under alkaline conditions, a simple mixture of catechol-conjugated Chi and PluSH (Chi-C/PluSH) achieved instant gelation at physiological temperature and pH, with hydrogels revealing enhanced strength due to intermolecular crosslinking between the two polymers. Moreover, wet adhesion experiments (performed in a universal testing machine—UTM, using mouse subcutaneous tissue) revealed higher detachment stress values for Chi-C/PluSH (~15.0 kPa) compared to controls lacking catechol (~5.3 kPa) or thiol (~6.6 kPa) groups, thus suggesting a role for catechol in the hydrogel adhesion to tissues and in the interaction with thiol groups (quinone–thiol bond) for the formation of stable hydrogels. Importantly, whereas Pluronic hydrogels were found to quickly erode, Chi-C/PluSH hydrogels exhibited significant stability in vitro (in aqueous solution) and in vivo (upon subcutaneous implantation in mice).

In another study, Ryu et al. [15] reported the development of an alternative method for the crosslinking of hydrogels that avoids the use of enzymes (e.g., horseradish peroxidase (HRP) or Tyr) or high concentrations of oxidating agents, based on a water-soluble enzyme-mimetic biocatalyst (hematin, Hem). In this regard, hydrogels were obtained within 5 min of mixing hematin-grafted Chi (Chi-g-Hem) with EDC-mediated HCA-functionalised Chi (Chi-C), with crosslinking catalysed by Hem under mild physiological conditions. Wet adhesion experiments (performed in a UTM using mouse subcutaneous tissue) revealed increased adhesive force for Chi-g-Hem/Chi-C hydrogels (~33.6 kPa) compared to those formed by pH-induced catechol oxidation (~20.6 kPa). Similar observations were reported for catechol-containing Chi-g-Hem-catalysed HA-C hydrogels (compared with untreated HA) and biocatalyst-induced polyvinyl alcohol (PVA)-C hydrogels. Importantly, the existence of optimal windows for hydrogel gelation times and polymer concentrations indicated that unreacted catechol residues (i.e., those not involved in the crosslinking network) contributed to the increased adhesion properties of the hydrogels.

Zhou et al. [22] explored the use of Chi and ε-polylysine (PLL) as adhesives for peripheral nerve regeneration, a field in which the current clinical options, cyanoacrylates and fibrin glue, have shortcomings that limit their application. The authors designed a nerve-adhesive hydrogel formed in situ and composed of a polysaccharide (Chi) and polypeptide (PLL), mimicking the polysaccharide/protein structure of natural epineurium matrices, which crosslinked via Michael-type addition between maleimide (PLL) and thiol (Chi) reactive groups. To improve the adhesivity of the hydrogel, catechol groups were conjugated onto the PLL backbone using standard EDC chemistry. Gelation occurred within 10 s and was not significantly influenced by the introduction of catechol groups. A lower catechol DS (5%) favoured the simultaneous improvement of the cohesive force of the hydrogel (storage modulus elevated to more than 2.4 kPa) and the interfacial adhesive force between the hydrogel and epineurium (>0.185 N of force, eightfold higher than that of fibrin glue). In vivo testing revealed better nerve regeneration performance of the hydrogel compared to current nerve surgery methods, with data at 8 weeks showing the morphology of the repaired nerve fibre coated by the hydrogel close to the morphology of normal nerve, and the axon cross ratio of the regenerated nerves coated using hydrogel (57%) higher than that using the suture technique (35%). Finally, in vitro and in vivo compatibility tests confirmed good nerve biocompatibility and low immunogenicity of hydrogels towards the nerve.

Aiming at developing new formulations for wound dressing and tissue adhesion, Park et al. [18] described the optimisation of catechol conjugation to CChi via two different functionalisation strategies: carbodiimide (EDC/NHS) chemistry (C-CChi) and enzymatic oxidation mediated by tyrosinase (Tyr) activity (E-CChi). For C-CChi synthesis, the carboxylic group of HCA reacted with free amines on Chi in the presence of EDC, whereas for E-CChi, a two-step protocol was performed, with initial Tyr-mediated conversion of HCA to o-quinone and subsequent reactions of this intermediate with amines of Chi. Grafting of catechol to the Chi backbone was confirmed by NMR analysis, whereas DS was calculated from UV–Vis measurements at 280 nm (8–27%). Structural properties of oxidatively-crosslinked CChi hydrogels were proportional to the degree of oxidation, i.e., constructs with increased degrees of oxidation presented denser microstructures, increased porosity, and smaller pore sizes. When mechanical properties were analysed by lap shear adhesion tests, the biomaterial adhesiveness did not change with increasing catechol DS for C-CChi and E-CChi. However, both formulations displayed higher lap shear strength compared to that of unmodified chitosan (at pH 7 and 8) and other catechol-modified Chi, as well as that reported in the literature for fibrin glue (~20 kPa). In vitro studies with NIH3T3 mouse fibroblast cells demonstrated that C-CChi and E-CChi hydrogels do not impact cell viability; higher cell adhesivity was observed for E-CChi in platelet adhesion studies. As the better-performing formulation, E-CChi was tested in an in vivo wound healing assay, with results revealing better healing performance for E-CChi hydrogels compared to the commercially available Dermabond^®^ adhesive, as well as similar healing performance, higher epidermis epithelialisation and collagen deposition in the dermis, compared to a standard suture procedure.

Shi et al. [23] described the development of a hybrid hydrogel system, composed of 3,4-dihydroxyphenyl propionic acid (DPA)-functionalised Chi and dopamine (DA)-modified gamma-polyglutamic acid (γ-PGA), to be used as an adhesive for biomedical applications. Functionalisation of the Chi involved preliminary activation of DPA with NHS and EDC (1:1:1), followed by mixing with a solution of Chi, whereas γ-PGA was activated with NHS and EDC (3:0.1:0.1) before the addition of DA. Hydrogels were then prepared by mixing the two solutions at different volume ratios. In this regard, fast electrostatic-mediated gelation kinetics were observed immediately upon mixing of the two solutions, with G’ ≈ G’’ for t < 10 s and G’ > G’’ for t > 10 s (i.e., the formation of hydrogel with a viscoelastic profile). Adhesion tests on material surfaces (formaldehyde resin, polyurethane, and aluminium) revealed increased adhesion strength with increased catechol content, independent on the substrates, with higher values obtained in formaldehyde resin. Importantly, high adhesion strength was also obtained in lap shear tests performed in biological substrates, with values reported for fresh porcine skin (25 kPa) and arthrodial cartilage (145 kPa) significantly larger than those reported for fibrin glue (10 kPa). Degradation of the bioadhesives, evaluated by measuring the weight change after incubation in PBS (pH 7.4, 37 °C), revealed initial stability of the constructs (20% weight loss after 1 day) followed by quick degradation (98% weight loss) over 1 week. Finally, in vitro biocompatibility studies showed reduced cytotoxicity (>90% cell viability) for L-929 mouse fibroblast cells incubated with hydrogels.

In addition to general tissue adhesives, catechol-modified Chi has been tested for application/delivery in oral and intestinal mucosa. Xu et al. [13] developed a buccal drug delivery system using a novel catechol-functionalised Chi hydrogel. Covalently bonded catechol groups were attached to the backbone of Chi by reaction with HCA, and crosslinked to the polymer with genipin. The gelation time and the mechanical properties of catechol-functionalised Chi hydrogels were similar to those of Chi-only hydrogels but the catechol groups significantly enhanced mucoadhesion in vitro (7/10 catechol-functionalised Chi hydrogels were still in contact with the porcine mucosal membrane after 6 h, whereas all Chi hydrogels lost contact after 1.5 h). The improved adhesive hydrogels were able to sustain the release of an anaesthetic drug in the mouth over longer periods of time.

Reaction with HCA was also used by Kim et al. [14] for the introduction of catechol groups onto Chi, generating polymers (Chi-C) with various degrees of catechol conjugation (7.2%, 12% and 20.5%). Modification with Chi, confirmed by surface plasmon resonance spectroscopy, resulted in enhanced mucoadhesion (over fourfold) compared to the unmodified polymer. Moreover, the retention in the intestinal mucosal layer of Chi-C (~10 h) was significantly longer than that of Chi or the widely used anionic mucoadhesive polymer poly(acrylic acid) (PAA, Mw 450 kDa), which were rarely detected 3 h after oral administration in mice. Importantly, the authors demonstrated that the improved gastrointestinal retention of Chi-C resulted from the formation of irreversible catechol-mediated crosslinking with mucin.

Striving to establish an effective treatment for oral mucositis, Ryu et al. [20] developed a Chi-based oral patch, modified with HCA for enhanced mucoadhesion. Functionalisation of the Chi backbone with catechol moieties (Chi-C) was mediated by EDC (DS = 6.5%, without catechol oxidation), with patches then prepared by freeze-drying of the Chi-C solution, creating porous, sponge-like constructs. Time-sweep measurements of Chi-C showed rapid dissolution of the patches in distilled water, with the addition of saliva quickly generating a high elastic modulus. Moreover, lap shear tests performed in porcine keratinised and non-keratinised mucosa revealed higher detachment forces for Chi-C (10.3 kPa and 20 kPa, respectively), compared to unmodified Chi patches (4.1 kDa and 3.2 kPa, respectively), thus indicating that interpolymer complexation mechanisms with mucosal macromolecules were mediated by catechol. Indeed, further testing confirmed the interaction of mucin and catechol groups to significantly improve the tissue adhesion force and stability of Chi-C patches in the oral cavity. Using triamcinolone acetonide (TA) as a model drug, release studies revealed an initial burst release (up to 4 h) followed by a slower sustained TA release. Importantly, the sustained release of Chi-C-formulated TA (25 μg) accelerated epithelial regeneration in severe oral ulcers, compared to TA-loaded Chi-C (12.5 μg), Chi-C or a commercially available adhesive.

Aiming at developing mucoadhesive biomaterial-based carriers for the treatment of oral cancer, Pornpitchanarong et al. [21] fabricated dual-polymer nanoparticles (NPs), functionalised with catechol (Cat) moieties and encapsulating doxorubicin (DOX), for strong adhesion and sustained drug release at the tumour site. For the preparation of NPs, Chi and hyaluronic acid (HA) were modified with DA, with Chi functionalisation involving a two-step reaction (i.e., the introduction of succinyl groups followed by EDC/NHS-mediated coupling of DA, to form SChiCat), while the same coupling chemistry was used for the direct modification of HA with DA (Hacat). Cat-NPs were then prepared via the ionic gelation of SChiCat and Hacat, under strong stirring and probe sonication. Dynamic light scattering (DLS) and scanning electron microscopy (SEM) analysis revealed the formation of negatively charged spherical structures (160 to 305 nm), with smaller and more homogeneous NPs obtained upon the mixing of SChiCcat and Hacat at a 2:1 ratio. Non-covalent DOX loading onto the best-performing Cat-NPs was found to be proportional to the drug concentration, with the highest loading efficiency (~70%) for a Cat-NPs:DOX ratio of 1:0.5, which was associated with fast DOX release (over 3 h) followed by slower release over 24 h. In an ex vivo porcine flow-through study, it was demonstrated that >60% of Cat-NPs remained attached to buccal mucosa after rinsing with artificial saliva, compared to control SChi/HA NPs (~50%) and dextran (10%), confirming that catechol functionalisation contributes to stronger mucoadhesive properties. In vitro studies in human gingival fibroblasts (HGF) and NH22 oral squamous carcinoma cells confirmed the biocompatibility of Cat-NPs (>80% cell viability after incubation for 24 h), whereas increased rates of uptake, accumulation and apoptosis were found for DOX-loaded Cat-NPs (compared to free DOX).

Innovating on the field of cancer therapy, Wang et al. [19] described the development of a new bioinspired manganese dioxide (MnO_2_) hybrid hydrogel (BMH) for simultaneous melanoma therapy, tissue regeneration and anti-bacterial activity. For this purpose, 2D anisotropic MnO_2_ nanosheets were combined with caffeic acid (CA)-modified chitosan (ChiCA, DS = 12.5%). MnO_2_-mediated crosslinked hydrogels exhibited a complex 3D porous structure with embedded black dots corresponding to the MnO_2_ nanosheets. Favourable adhesive properties were evident after adhesion of hydrogels to the finger knuckle, as well as in lap shear mechanical tests, which showed increasing hydrogel adhesiveness with increasing polymer or catechol content (up to 3.35 kPa), although not significantly different from that of fibrin glue. BMH hydrogels also demonstrated self-healing capacity when subjected to cycles of intercalating strains and shear thinning behaviour, making them suitable for wound dressing applications. The presence of MnO_2_ nanosheets not only led to crosslinking, but allowed (i) catalytic conversion of H_2_O_2_ into O_2_, to mitigate the hypoxic tumour microenvironment, (ii) controlled spatiotemporal release of DOX, and (iii) photo-mediated increases in the local temperature (up to 49 °C), leading to the induction of apoptosis and extensive cell death of A375 melanoma cancer cells. Importantly, testing of BMH hydrogels, carried out in two different disease models, revealed (i) >95% growth inhibition of cancer cells in an in vivo subcutaneous melanoma mouse model, and (ii) accelerated closure and re-epithelialisation of the wound site in a microbial-infected full-thickness murine cutaneous wound model.

##### Hyaluronic Acid (HA)

HA is an anionic non-sulphated glycosaminoglycan, composed of repeating disaccharide units of D-glucuronic acid and N-acetyl-D-glucosamine. Present in most connective/epithelial tissues, and abundant in human joints, HA molecular weight is proportional to the number of repeating monomers, with values ranging between 4 kDa and 8000 kDa. Several manuscripts described the functionalisation of HA with catechol moieties for application in different biomedical areas requiring adhesivity, including tissue regeneration, wound healing and biosensing.

In a well-designed study, Koivusalu et al. [28] focused on the development of novel adhesive and implantable biomaterials for the transplantation of distinct cell populations to the cornea, aiming at achieving corneal regeneration. Using established carbodiimide coupling strategies, the authors synthesised DA-grafted HA via the activation of carboxyl groups with EDC (DS = 14%), while a similar approach was used for the conjugation of carbodihydrazide (CDH) to HA or HA-DA (DS = 10%). Hydrogels were produced by the combination of equal volumes of HA-CDH/HA-DA-CDH and aldehyde-modified HA (DS = 10%), using conventional hydrazone crosslinking chemistry. Rheological profiling of DA-functionalised hydrogels and control constructs (without DA) showed G’ > G’’ for the screened frequencies, indicating the formation of stable, viscoelastic-like hydrogels with strong elastic character (G’/G’’~0.003). Although both hydrogels showed similar swelling behaviour, the kinetics were dependent on the surrounding buffering conditions, with those incubated in PBS reaching equilibrium after 1 week, whereas a sustained linear increase was found for those incubated in culture medium. Furthermore, analysis of hydrogel adhesion to porcine corneal tissue (via tack adhesion tests) revealed greater adhesion strength for DA-modified hydrogels. Importantly, tissue-like cellular compartmentalisation was achieved by the encapsulation of hASCs inside the hydrogels and the seeding of limbal epithelial stem cells (LESC) in the hydrogel surface, which was previously modified with thiolated basal membrane proteins. The compartmentalised DA-modified hydrogels not only allowed the maintenance of cell phenotype and functionality, but displayed excellent tissue adhesion upon sutureless implantation in a porcine corneal organ culture model.

Similarly, Shin et al. [25] successfully synthesised HA–catechol conjugates (HA-Cat), with adhesive and cohesive properties, for minimally invasive cell-mediated liver regeneration. HA-Cat was produced by the conjugation of DA to the HA backbone via EDC/NHS-mediated carbodiimide coupling reactions (DS = 4–10%), with gelation achieved via the oxidative crosslinking of catechol groups using NaIO_4_ under alkaline conditions. Compared to a widely used photo-crosslinkable methacrylate HA (HA-ME), fabricated by the transesterification of HA with methacrylate anhydride (DS = 9.8%), HA-Cat presented (i) greater swelling capacity, (ii) faster degradation (i.e., 50% weight loss after incubation for 2–4 h in hyaluronidase-containing buffer), and (iii) lower storage moduli (1321 vs. 3443 Pa), indicative of softer hydrogels, with a stable viscoelastic profile (G′ > G’’) confirmed by measurements in the entire frequency spectrum. Importantly, testing of hydrogel adhesivity to liver tissue, using a UTM, revealed the superior adhesion strength of HA-Cat hydrogel (~1.4 KPa, DS = 8.8%) compared to that of HA-ME hydrogels (~0.19 KPa, DS = 9.8%), which was directly proportional to the degree of catechol substitution. In addition to improving the viability and function of encapsulated hepatocytes and adipose-derived human mesenchymal stroma-like cells (hASCs), and enhancing in vivo attachment to liver and heart tissues, HA-Cat hydrogels enabled the minimally invasive transplantation of hepatocytes to the liver, with constructs supporting the adhesion, proliferation and biological activity of the transplanted hepatocytes.

Aiming at developing innovative hydrogel-based wearable devices for human motion monitoring, with optimal stretchability, conductivity and self-adhesive properties, Lv et al. [27] designed a tri-component system featuring DA-modified HA (HAC), acrylamide (AM) and borax (as a dynamic cross-linker), as well as the conductive cations lithium and sodium. HAC was synthesised by a reaction of HA with carboxyl-activating agents EDC and NHS, followed by the addition of DA (DS = 34%, without the oxidation of catechol). Optimisation of reaction conditions included fine-tuning the HAC/AM ratio, with values >6% (wt.%) preventing hydrogel formation, because the reduced catechol groups delayed AM polymerisation. Stable hydrogels, supported by catechol, hydroxyl, and amine groups present in the HAC chains and surrounding network, which form interchain and intrachain hydrogen bonds, were produced, with mechanical and electrical tests revealing enhanced stretchability (2870%), swelling ability, and high tensile toughness (42 kPa). Moreover, the catechol groups in HAC enhanced (i) self-adhesiveness, as demonstrated by the strong adhesive strength of the constructs to porcine skin (49 kPa), and (ii) efficient self-healing without stimuli at room temperature, as reflected by quick wound healing times (~1 h). Reduced conductivity was detected for HAC-B-PAM hydrogels (0.018 S/m), whereas increased values were found for HAC–B–PAM hydrogels containing lithium (1.10 S/m).

For the development of HA-based hydrogels with improved adhesive properties and stability, Zhou et al. [26] devised a strategy for the preparation of DA-functionalised HA hydrogels with enhanced catechol content, based on Schiff’s reaction of DA with aldehyde-modified HA. Compared to carbodiimide-based coupling strategies (average DS ~10%), higher DS was found for the coupling of DA to dialdehyde-modified HA (DAHA) (25% to 45%), without the formation of polydopamine or oxidation of catechol. The kinetics of oxidative crosslinking and gelation were assessed by time sweep rheology, with measurements revealing fast gelation for catechol-functionalised DAHA (CatHA) constructs (G’ = G’’ under 60 s), and G’ > G’’ until complete stabilisation of the hydrogels (at ~8 min). CatHA hydrogels presented a porous interconnected structure (20−350 μm pores) and significant swelling capacity (15–40 times greater than their initial weight), which was inversely proportional to the DS of DA (i.e., smaller pores and lower swelling capacity were associated with a denser structure characteristic of higher DS). Adhesion of CatHA hydrogels to porcine skin, measured via lap shear tests, was found to be directly proportional to the catechol content of the constructs, with higher values obtained for CatHA (~90 kPa), compared to conventional DA-conjugated HA hydrogels (~10 kPa), UV-crosslinked HA hydrogels (~13 kPa) and fibrin glue (2–40 kPa). Importantly, biocompatibility assays performed in L-929 cells revealed no significant cytotoxicity of the constructs (metabolic activity > 80%), which were found to support cell adhesion and proliferation.

Similarly, Zhang et al. [29] described a novel method for the preparation of catechol (Cat)-functionalised HA, involving the conjugation of Cat functional groups to the HA backbone in organic phases under a nitrogen atmosphere, to increase the DS and prevent the oxidation of catechol. By reacting DA with the carboxyl groups of HA through carbodiimide/N-hydroxybenzotriazole (HOBt) coupling chemistry, the authors reported an average DS of 33% (compared to 7% for synthesis with the conventional method), without the detection of UV peaks characteristic of oxidised catechol. Following simple oxidant-mediated gelation, stable HA-Cat hydrogels were formed in ~20 min, with high DS correlating with faster gelation times. Similarly, wet adhesion testing of HA-Cat or control catechol-free HA hydrogels in chicken skin revealed a higher adhesion strength for HA-Cat hydrogels (compared to those without catechol or commercially available fibrin glue), which was directly proportional to the catechol content. Importantly, extensive cell adhesion and spreading were observed for human mesenchymal stem cells (hMSCs) seeded on the surface of the HA-Cat hydrogels, with cells presenting a typical spindle-like morphology, whereas very few cells adhered and expanded on the surface of control catechol-free hydrogels. Moreover, metabolic assays confirmed that hMSCs encapsulated in HA-Cat hydrogels remain metabolically active, at levels similar to those of well-established catechol-free hydrogels. Finally, studies with BMP-2-laden HA-Cat hydrogels demonstrated the capacity of the constructs to promote osteogenic differentiation of the encapsulated hMSCs, via sustained release of the therapeutic cargo.

Sousa et al. [24] used a versatile and robust layer-by-layer technique to developed bioinspired films for wound-dressing, combining (via electrostatic interaction) Chi, alginate and HA modified with DA via carbodiimide chemistry (HA-DA, DS = 24%). SEM images revealed that the surface of the DA-containing membranes was more porous than the control unmodified counterparts. Moreover, DA-modified membranes showed higher average thickness values (for the same number of layers), improved mechanical strength and enhanced adhesion (assessed by lap shear strength test) compared to unmodified controls. The adhesion and proliferation of human dermal fibroblasts were also improved by DA-modified membranes, whereas treatments of dermal wounds in rats with DA membranes resulted in the strongest decrease in skin inflammation, compared with control conditions.

##### Silk Fibroin (SF)

Fibroin is the main component of silk, providing the internal structure and mechanical strength, with sericin forming an outer glue-like coating. Due to its excellent mechanical properties (as result of the formation of microfibrils with strong interlocks) and high biocompatibility, silk has been tested for use in several biomedical applications.

In this regard, Sogawa et al. [31] reported on the adhesive profile of SF, modified to express dihydroxyphenylalanine (DOPA) via the environmentally friendly enzymatic (Tyr) conversion of tyrosine. The content of DOPA was evaluated by amino acid composition analysis, with results showing an average DS of 1.3 mol%, which was generally constant for all the tested concentrations, and a tyrosine conversion ratio of ~25%. The adhesive strength of DOPA-modified SF (DOPA-SF) towards soft and hard material surfaces, including mica, paper, polypropylene, wood and silk film, were assessed by lap shear tests, with results for the mica substrate showing an improvement in both adhesive strength and fracture upon the introduction of DOPA at any silk concentration and pH level (maximum values of 0.97 MPa and 19.1 kJ/m^3^, respectively, at pH 10). Moreover, Fourier-transform infrared (FTIR) measurements demonstrated that the adhesivity of DOPA-SF was not directly related to the formation of secondary β-sheet structures, but to the catechol content. Importantly, DOPA-SF exhibited stronger adhesivity to all tested substrates (compared to unmodified SF), thus confirming the added value of DOPA functionalisation towards the improved adhesion of SF.

Using a similar enzyme-based strategy, Heichel et al. [32] described an improved approach for the in situ functionalisation of SF with catechol groups, towards its application in wound healing. Using a multi-step protocol, the authors initially modified purified SF by carboxylation, to increase the carboxylate content of SF for reactions with tyramine. Conjugation of tyramine to SF, via COMU or CDI coupling, enabled the enrichment of SF in phenolic side chains (~30% phenolic addition for both methods), which were further oxidised into catechol groups via mushroom Tyr. Rheological time sweeps performed at 37 °C revealed that formation of hydrogels with elastic profiles (G’ > G’’ for all frequencies), with faster gelation kinetics for conjugates containing tyramine or higher SF concentrations. Modification of SF with tyramine did not prevent the formation of secondary beta sheet structures within SF, thus allowing for noncovalent interactions to strengthen the structure. Single lap shear tests measuring the adhesion of SF conjugates to porcine intestines revealed enhanced adhesivity for tyramine–SF conjugates, compared to that of SF without tyramine or fibrin glue, without significant differences between tyramine(CDI)-SF and tyramine(COMU)-SF (due to similar DS). Moreover, the induction of beta-sheet secondary structures (via sonication) further improved adhesivity, thus suggesting that, in addition to enzymatic (covalent) crosslinking, physical (non-covalent) crosslinking of the constructs provides extra strength and adhesiveness. Finally, in vitro biocompatibility assays in CaCO-2 intestinal epithelial cells, assessing the toxicity of SF extracts, cell attachment and proliferation, revealed no significant toxicity from possible leachables.

To balance its hydrophobicity, SF was modified by Burke et al. [30] via the conjugation of hydrophilic polyethylene glycol (PEG) side chains (5 kDa), prior to reactions with DA for the chemical coupling of catechol groups. Although the efficiency of catechol functionalisation was not affected by the degree of PEG substitution, fine-tuning PEG modification was essential for aqueous solubility. Measurement of the adhesive bond of the modified silks to aluminium shims, using single lap shear testing, revealed that DA-modified SF formed a stronger adhesive bond compared to the unmodified material (>20 kPa improvement). Interestingly, the authors showed that incorporation of as little as 6 wt.% PEG prior to catechol functionalisation resulted in complete aqueous solubility of the catechol conjugates and increased the adhesive strength compared to silk lacking catechol moieties. PEG-SF conjugates also maintained the ability to form β-sheet secondary structures, which can be exploited to reduce swelling. Finally, in vitro biocompatibility tests revealed that the modified and unmodified SF supported the attachment and proliferation of hMSCs for up to 15 days, regardless of PEG and catechol conjugation.

Liu et al. [33] reported the development of novel SF-based bioadhesive hydrogels. For this purpose, the authors modified purified SF with catechol functional groups through a one-step cross-linking protocol involving NHS/EDC activation and subsequent DA loading (NESFB-DA, DS = 0–15%, without catechol oxidation). For gelation, different amounts of PEG (Mw 20 kDa) were added to NESFB-DA, to obtain pegylated NEFSB-DA hydrogels. The optimal ratios of SF:PEG (5:5), NHS:EDC (2:1), and the amount of DA (5 wt.%) were identified after fine-tuning of the reaction conditions. As revealed in dry adhesive testing, increasing the SF:PEG ratio from 5:1 to 5:5 (*w/w*) resulted in over a fourfold increase in the tensile strength of NESFB-DA hydrogels (from 48 kPa to 217 kPa), while decreasing the time required for hydrogel formation (from 116 min to 10 min). Importantly, adhesive tests in wet conditions (using fresh porcine skin) confirmed that pegylated NESFB-DA hydrogels display higher tensile strength compared to those without DA or clinically used fibrin sealants, which highlights the important role of DA in the adhesive properties of the hydrogels. Finally, degradation studies revealed a slow degradation rate for NESFB-DA constructs in PBS buffer (91% remaining weight after 55 days) and a better degradation profile compared to NESFB.

##### Gelatine

Gelatine is composed of a mixture of polypeptides produced by the hydrolysis of collagen. It is commonly used as a biomaterial for cell culture applications due to its biocompatibility, degradability and similarity to the extracellular matrix (ECM) at chemical and biological levels. In this regard, several adhesive gelatine-based constructs have been described for applications in wound healing and tissue regeneration.

Aiming at developing a new low-cost sealing materials with improved functional properties based on Gelfoam (an FDA-approved non-adhesive gelatine sponge for haemostatic applications), Hong et al. [36] modified gelatine (Gel) by conjugation with DA via an EDC coupling reaction, resulting in a modified polymer (GelDA) with ~7–8 catechol groups per backbone unit. Fe^3+^ complexation of Gel or GelDA yielded hexavalent Fe^3+^ complexes with metallo-bioadhesive properties. The adhesive strength of Gel/Fe and GelDA/Fe was measured by lap shear testing on porcine skin under natural, moist conditions, with results revealing better tissue sealing performance for GelDA/Fe over a wide range of FeCl_3_ concentrations (50–1000 mM), and especially at lower concentrations (over a twofold improvement in adhesive strength versus Gel/Fe for FeCl_3_ concentrations between 50 and 200 mM). Upon subcutaneous implantation in mice, both materials were completely degraded after 23 days, with GelDA/Fe showing a longer half-life (7 days) compared to Gel/Fe (2 days), possibly due to the covalent nature of the crosslinking induced by Fe^3+^.

An innovative approach was developed by Xuan et al. [38] for the treatment of acute traumatic wound healing. Based on material nanosheets, the authors designed a two-layer nanosheet, composed of DA-functionalised antimicrobial peptide (AMP)-modified gelatine and polycaprolactone (PCL), with strong mechanical properties, adhesivity and antimicrobial activity. Gelatine methacrylate (GelMA) was initially modified by conjugation with DA via EDC/NHS coupling chemistry, followed by functionalisation with thiolated AMP (via thiol-ene click chemistry). Composite nanosheets, containing UV-crosslinked GelMA/DA/AMP (GDP), Ca^2+^ and PCL, were manufactured by spin coating, producing thin films with final bilayer thickness of 45–95 nm and a rough PCL surface, which was smoothened after combination with GDP. Burst pressure and dynamic mechanical analysis confirmed that GDP/PCL constructs display suitable mechanical properties, including burst pressure superior to blood pressure and tensile modulus closer to that of human soft tissues. The flexible GDP/PCL nanosheets could also firmly attach to porcine tissue, although a gap was found with a more rigid control GDP hydrogel. Moreover, under wet conditions, GDP/PCL nanosheets remained attached to porcine tissue slices, whereas PCL nanosheets detached from the substrate, thus suggesting a role for catechol groups in the GDP on wet adhesion. Due to the incorporation of Ca^2+^ (a known activator of coagulation) in the GDP layer, the constructs were also found to promote rapid haemostasis, whereas the presence of AMP contributed decisively to strong antibacterial activity (~100% killing activity after 2 h), compared to GDP/PCL without AMP (<2% killing activity). Finally, studies in an in vivo mouse model of wound healing revealed the successful sealing of bleeding wounds upon applications of GDP/PCL, which was associated with reduced clotting time and blood loss, as well as an absence of strong inflammatory responses or microbial activity.

Wu et al. [37] designed a double hydrogel/patch system for the treatment of myocardial infarction (MI), for which single approaches have revealed limited therapeutic efficacy. Based on naturally occurring macromolecules, the authors developed a gelatine-based adhesive and conductive hydrogel patch, and an HA-based injectable hydrogel, for coadministration in the infarcted tissue. The injectable hydrogel was designed for in situ crosslinking via Schiff base reaction between oxidised sodium HA (HA-CHO) and hydrazided HA (HHA), whereas the self-adhesive cardiac patch was prepared via the ionic (Fe^3+^-mediated) crosslinking of gelatine−DA (GelDA) and DA-functionalised polypyrrole (DA−PPy). The proposed combinatorial approach involved injection of the HACHO/HHA hydrogel into the infarcted cardiac tissue, followed by layering of the GelDA/DA−PPy patch onto the outer surface of the myocardium, without the need for additional sutures. Adhesion tests performed in porcine myocardium and skin revealed significant catechol-mediated adhesion strength for GelDA/DA−PPy-0.6% hydrogels (10 kDa and 16 kDa, respectively), which could withstand twisting, bending and soaking in water, as well as conductivity values matching those of myocardial tissue (2.85 × 10^−4^ S/cm). Rheology tests with HA-CHO/HHA hydrogels (5% HA-CHO, 0.4−0.8% HHA, wt.%) revealed fast gelation kinetics and the formation of stable viscoelastic structures (i.e., G′ > G″ for the entire frequency range). Importantly, echocardiographical, histological and angiogenic outcomes were improved in rats, 28 days after surgical administration of the combined therapy, compared to those untreated or receiving single therapy.

Aiming at manufacturing small-diameter vasculature, containing a smooth muscle and endothelium, Cui et al. [39] developed a smart mussel-inspired bioink, based on catechol-functionalised gelatine methacrylate (GelMA/C), capable of forming elastic hydrogels upon oxidative in situ crosslinking, with controllable mechanical strength, enhanced adhesivity and tuneable physical cues for bioprinting of stratified architectures. GelMA/C was synthesised in a stepwise process, involving carbodiimide coupling reactions of porcine gelatine with methacrylic anhydride, followed by DA grafting via EDC/NHS coupling chemistry (DS = 6–13%). Physico-chemical characterisation of the hydrogels included analyses of rheological properties, swelling and adhesion. In this regard, the authors noted a catechol- and solution-concentration-dependent gelation time, with GelMA/C at >15 wt.% favouring rapid gelation compatible with 3D bioprinting, whereas the high viscosity of bioink solutions at >30 wt.% hindered the extrusion process. G’ > G’’ was found for GelMA/C hydrogels over the entire frequency range, with the swelling of constructs reaching equilibrium or saturation in 3 days, and the adhesion strength of GelMA/C significantly higher than that of the GelMA counterpart. Cell-laden 3D tubular constructs were produced by coaxial printing with a double-needle system, using 15 wt.% GelMA/C (DS = 13%), human coronary artery smooth muscle cells (HCASMCs) and human umbilical vein endothelial cells (HUVECs). Using a cross-printing process, a well-connected vessel network was obtained, with an inner porous structure that allowed continuous perfusion, as well as gas and nutrient exchange. Indeed, in vitro cell assays confirmed the maintenance of viability and proliferation of encapsulated HCASMCs and HUVECs after 7 days in culture, with beneficial permeability parameters measured in the vascularised channels. Importantly, in vivo evaluation of degradability and biocompatibility of the 3D-bioprinted vasculature, using a mice xenograft model of transplantation, revealed slow (but incomplete) degradation of the subcutaneously implanted constructs over 16 weeks, and an inflammatory response typical of foreign body responses.

##### Alginate

Alginate is a naturally occurring polysaccharide derived from brown algae, with wide uses in food and biomedical industries. Surprisingly, only a reduced number of manuscripts involving alginate complied with the selection criteria.

For the development of surgical polysaccharide-based membranes with improved adhesion, Sconamiglio et al. [34] functionalised alginate by grafting DA via an EDC/NHS coupling reaction between the carboxylic groups of the polymer and amine groups of DA, with the characterisation of the modified biomaterial focused specifically on its adhesive properties. In vitro adhesion studies, carried out to evaluate the adhesiveness of the membranes in simulated physiological conditions, revealed that the adhesivity of the target tissue (intestine serosa) was enhanced in the DA-modified alginates, with over 50% of the membranes containing high degrees of substitution (1.8% and 2.8% by NMR spectroscopy) still attached to the intestinal tissue after 6 h of complete immersion in deionised water. In vivo assessments of the adhesiveness of DA-modified membranes were performed in a porcine model, with results showing good initial adhesion for both DA-modified and control non-modified membranes in contact with the tissue, although at 7 h post-implantation within the pig abdomen, only the DA-modified membrane remained attached to the intestinal serosa. Early adverse tissue reactions upon contact with the DA-modified membrane could not be detected by histological analysis, thus highlighting the biocompatibility of the material after 7 h of implantation.

Aiming at understanding the impact of catechol modification on the mechanical properties, wet adhesive strength and contact behaviour with soft tissues, Cholewinski et al. [35] carried out studies with catechol-modified or unmodified alginate hydrogels, using gelatine as a model tissue-like material. Using hydrogel beads as the testing probe (which allows for testing of interactions with rigid and soft substrates), modified alginate gels showed poor adhesion to hard surfaces (namely glass and gold), although improvements were seen across protein-based substrates. The authors showed that a degree of catechol substitution <3% significantly improved the adhesion of alginate to gelatine by half an order of magnitude, in a time- and pH-dependent process (not detected in unmodified alginate), which is most likely the result of oxidation of the catechol group and/or reaction kinetics. Further tests indicated that the strong adhesion of catechol–alginate to gelatine substrate can extend to other protein-based substrates, such as animal tissue, thus highlighting the potential applications of alginate–catechol hydrogels as tissue adhesives or wound dressings.

##### Carboxymethylcellulose (CMC)

CMC is an anionic water-soluble cellulose derivative, composed of repeated glucopyranose monomers modified by the conjugation of carboxymethyl groups, which is widely used as a thickener, lubricant and pharmaceutical excipient. A couple of studies addressing improved adhesiveness of CMC were found to comply with the search/scoring criteria.

Fu et al. [44] designed a hybrid system, based on CMC, to be used as an underwater adhesion hydrogel. For this purpose, oxidised CMC (OCMC) was prepared using the periodate oxidation method, and subsequently modified by combination with DA via carbodiimide (EDC/NHS) crosslinker chemistry (average DS = 15%). OCMC-DA/PAM hydrogels were obtained upon the conjugation of acrylamide monomers (AM) to OCMC-DA via a Schiff base reaction, followed by the chemically induced polymerisation of AM. Structural/morphological and functional analysis of hydrogels included mechanical, rheological and adhesivity tests, as well as assessments of swelling behaviour and cytotoxicity. In this regard, the authors reported the formation of hydrogels with porous 3D structure, G’ > G’’ (at 0.1–50 Hz) and storage modulus increasing proportionally to the concentration of OCMC-DA. The adhesion strength of OCMC-DA6/PAM5 (one of the synthesised hydrogels) reached ~86 kPa (compared to 5 kPa for PAM5) and reduced to 43 kPa after immersion in water for 9 days. Moreover, the maximal adhesion strength was obtained when the G’ and G’’ of hydrogel were very close. Finally, no significant toxicity was found when NIH-3T3 mouse embryonic fibroblasts were incubated with extracts prepared from different OCMC-DA/PAM hydrogels.

Aiming at preparing mussel-inspired adhesive hydrogels for wound closure applications, Zhong et al. [45] prepared novel CMC-based constructs amenable to in situ enzymatic crosslinking. Following EDC/NHS-catalysed CMC functionalisation with catechol moieties, CMC-DA hydrogels were fabricated via horseradish peroxidase (HRP)-mediated crosslinking in the presence of hydrogen peroxide (H_2_O_2_). Extensive testing was performed to investigate the impact of HRP/H_2_O_2_/CMC-DA concentration and DS on gelation/degradation kinetics, swelling and mechanical properties. Spectroscopic quantification of catechol aromatic rings revealed a DS between 4.5% and 13.5%, which was proportional to the amount of reacting DA. The hydrogel crosslinking rate was affected differently by the HRP/H_2_O_2_/CMC-DA concentrations, including (i) decreasing the gelation time with increased HRP/CMC-DA concentration, and (ii) decreasing the gelation time with increased H_2_O_2_ concentration up to 5 mM. On the mechanics of in situ gelation and degradation, time sweep rheology measurements revealed a more elastic profile for hydrogels with higher catechol content, as result of a denser 3D structure, which was associated with slower cellulase-mediated degradation kinetics (40–60% weight loss over 1 week). Greater adhesivity was also detected for CMC-DA hydrogels with higher DS (up to 28.5 kPa), which was superior to that of commercially available fibrin glue (2–6-fold increase). Finally, normal cell morphology and viability >89% were detected for L-929 murine fibroblasts cells cultured on the surface of CMC-DA hydrogels.

##### Other Natural Polymers

For several other naturally occurring polymers, including polysaccharides such as chondroitin sulphate, dextran, xanthan gum and lignin, a single manuscript complied with the search and scoring criteria.

Shin et al. [42] designed a new hydrogel for cartilage repair based on the functionalisation of chondroitin sulphate (CS) with DA (CS-DA) using carbodiimide (EDC/NHS) chemistry. Conjugates with a DS around 20.9% showed (i) the least degradability when exposed to chondroitinase, (ii) the highest elastic modulus (~11 kPa), and (iii) the strongest adhesion to porcine cartilage (20−60 times greater than control methacrylated CS (CS-ME) hydrogels), thus suggesting a key role for catechol groups in hydrogel performance. CS-DA also showed catechol-related biocompatibility, by enabling high cell viability of encapsulated hASCs (>97% for all CS-DA groups on day 7) and presenting low immunogenicity, as per ELISA quantification of the pro-inflammatory cytokine tumour necrosis factor alpha (TNF-α) secreted by RAW264.7 macrophages. In vivo testing of the CS-DA hydrogels was performed in a dorsal subcutaneous rabbit model upon combination of the composites with printed PCL scaffolds (to provide stronger structure). In this regard, better cartilage formation and the higher expression of chondrogenic markers Sox9 and Col2A1 was found for CS-DA-PCL combined with autologous diced rabbit cartilage, compared to those without diced cartilage. Following implantation in an auricular cartilage tissue transplantation model, CS-DA-PCL hydrogels containing ear cartilage attached tightly to the wound edge, while similar CS-ME-PCL hydrogels detached from the defect, thus confirming the role of catechol moieties in the adhesion of CS-CA hydrogels to tissue.

Aiming at improving the adhesive profile of PEG:dextran hydrogels for use as surgical sealants, which are significantly affected by material swelling around soft tissues, Shazly et al. [43] modified star PEG amine and dextran aldehyde polymers by the covalent conjugation of L-DOPA to linear dextran aldehyde using dynamic imine (amine–aldehyde) chemistry. Functional analysis of hydrogels, formed by spontaneous reaction upon mixing of the two components, included mechanical testing (ex vivo burst pressure assays in rat intestinal wounds), analysis of swelling and compressive modulus, as well as evaluations of local immune responses upon subcutaneous implantation in mice. In this regard, results clearly demonstrated overall improved performance for DOPA-modified PEG:dextran hydrogels (at 3 mM DOPA/M of aldehyde), including (i) lower swelling (50.3% less), (ii) higher stiffness (>280% increase), and (iii) stronger adhesive wound closure strength (>50%) compared to control unmodified hydrogels. However, although higher concentrations of DOPA (up to 11 mM/M of aldehyde) were similarly effective in reducing swelling, the adhesive strength and biocompatibility of the hydrogels were significantly reduced. Indeed, thicker fibrous layers were found surrounding conjugates containing 8 mM and especially 11 mM DOPA (compared to minimal fibrosis for unmodified PEG:dextran implants).

Aiming at addressing the poor healing of postoperative anastomotic leakage (PAL), a severe complication of gastrointestinal surgery, Huang et al. [41] developed a novel hydrogel formulation composed of xanthan gum (Xan) modified with DA via EDC/NHS coupling (DS = 4% to 16%), to obtain a functionalised Xan hydrogel (DA-g-Xan) with improved adhesive properties. The combination of moderately adhesive Xan with DA was found to be sufficient for the induction of hydrogel crosslinking, due to the abundant intermolecular hydrogen bond interactions. Indeed, gelation could be easily observed upon inversion and shaking of the hydrogels, with DA (in 5 wt.% DA-g-Xan) providing additional structural integrity to resist centrifugation forces, whereas 5 wt.% Xan was destroyed by centrifugation. These results were corroborated by (i) rheological analysis, revealing that G’ increases with increasing polymer concentration, with DA grafting further improving G’ for each polymer concentration, and (ii) lap shear strength tests, showing a synergistic effect between Xan and DA adhesiveness towards the overall adhesive strength of DA-g-Xan, with values surpassing those of the commercially available fibrin glue. Interestingly, DA-g-Xan hydrogels presented self-healing properties but quick degradation in vitro (i.e., almost no biomaterial remaining after incubation for 96 h at 37 °C in PBS). On what concerns the influence of the biomaterial on cell activity, an anti-inflammatory profile was detected upon the incubation of RAW264.7 macrophages with DA-g-Xan (20 µg/mL) for 24 h, which included the overexpression of IL-4, IL-10 and CD206. Importantly, DA-g-Xan hydrogels improved the healing of colonic anastomosis in a rat model of disease, thus proving to be an approach with clinical potential.

Inspired by the mechanisms of adhesion in mussels, Gan et al. [46] designed tough, adhesive and antibacterial hydrogels based on plant-derived polymers. To achieve the necessary redox balance, the authors built a system for the continuous generation of catechol groups driven by reductive phenolic/methoxy groups present in lignin, which are capable of reducing silver ions (Ag^+^) to metallic silver nanoparticles (Ag-NPs) while oxidising to the corresponding o-quinone. Photogenerated electrons produced by Ag-NPs are then responsible for the conversion of o-quinone to catechol. In this regard, hydrogels with optimal mechanical properties were produced by the combination of Ag-lignin NPs with pectin and polyacrylic acid (PAA), which creates, upon gelation at room temperature (mediated by ammonium persulfate), an internal network of covalent and non-covalent bonds endowing high hydrogel toughness. The continuous and dynamic generation of catechol moieties by Ag-lignin NPs in the inner redox hydrogel network allowed for repeatable adhesiveness; the pectin/PAA mesh provided significant stretching capacity (>26 times its initial length) and quick recovery after deformation. Strong long-lasting adhesiveness was also measured for hydrogels in various surfaces, including glass, titanium, polytetrafluoroethylene and porcine skin (38, 50, 65, and 27.5 kPa, respectively), with further experiments suggesting a role for carboxyl groups of PAA and catechol groups of Ag-lignin in the molecular mechanisms of adhesivity. Importantly, in vitro and in vivo studies in a rabbit model clearly revealed strong antibacterial activity of the developed hydrogels, thus highlighting their application potential in wound healing.

#### 3.1.2. Synthetic Polymers

Compared to naturally occurring biomaterials, which are more abundant and biologically resemblant of the extracellular milieu, synthetic polymers provide significant versatility, while offering improved physicochemical consistency. In the context of this literature review, synthetic polymers accounted for a smaller percentage of the final list of references (13/47, ~28%), headlined by polyethylene glycol and cyanoacrylate derivatives, with most studies describing the development of improved tissue adhesives/sealants (see Table 4).

**Table 4:** Studies involving synthetic polymers, selected for inclusion.

Modified Polymer	Substituent/ Coupling Chemistry	Crosslinking Mechanism/ Catalyst	Adhesive Strength	Reported Features/Findings	Intended Application	Ref.
Poly(ethyleneglycol)	DA/NHS-mediated amine-reactive chemistry	Oxidative/NaIO_4_	8 kPa	Adequate curing rate Biocompatible (in vitro)	Tissue engineering and drug delivery	[47]
	L-DOPA/carbodiimide chemistry	N.A.	50 ng/cm^2^ (mucoadsorption) and 356.108 pN (pull-off force)	New simple approach for rapid screening of mucoadhesive polymers	Drug delivery	[48]
	L-DOPA/carbodiimide chemistry	Oxidative/NaIO_4_	35 ± 12.5 kPa	Fast gelation	Tissue adhesive	[49]
	DA/Michael Reaction	Photo-mediated (UV light)	6.1 ± 0.5 MPa	Rapid curing rate Biocompatible (in vitro)	Tissue adhesive	[50]
Prepolymerised allyl 2-cyanoacrylate	L-DOPA or DA/N.A.	Chemical (Michael addition)	0.71 ± 0.04 MPa	Improved mechanical properties Biocompatible (in vitro)	Bio-glue	[51]
Poly(dopamine methacrylamide-co-Methoxyethyl acrylate)	DA/Messersmith’s method	Ionic/CaSO_4_ and MgSO_4_	165 ± 13.5 kPa	Increased adhesion with PDMC content in the blend	Tissue adhesive	[52]
Pluronic F127	DA/activation by p-NPC and reaction with DETA or TETA	Chemical (Michael addition)	13.7 ± 1.6 kPa	Stable at physiological conditions Biocompatible Fast gelation (10 s)	Tissue adhesive	[53]
Recombinant fp-1 (rfp-1) mussel adhesive protein	L-DOPA/enzymatic reaction (tyrosinase)	Oxidative or Ionic/NaIO_4_ or Fe^3+^	200 kPa (ox. gelation) and 130 kPa (ionic gelation)	Flexible viscoelastic behaviour Self-healable	Tissue adhesive	[54]
Thiourea-linked monoacrylated β-cyclodextrins	Catechol-RGD/post-gelation functionalisation via thiourea-catechol coupling reaction	Photo-mediated (UV light)	2.0 kPa	Tuneable gelation, dynamic modulus and physical properties Stretchable (up to 17 times its original length) Biocompatible (in vitro)	Tissue adhesive and sealant	[55]
Polymethacrylic acid	L-DOPA, carbodiimide chemistry	Solvent exchange	1780 mJ/m^2^	Decreased hydrophilicity and increased adhesiveness with substitution	Tissue adhesive	[56]
Ureido-pyrimidinone	DA/HATU chemistry	Solvent casting (films)	170.9 ± 13.4 cell/mm2	Biocompatible (in vitro)	Cell therapy, tissue engineering	[57]
Polyacrylic acid and alginic acid	Adenine-DA/carbodiimide chemistry	Oxidative/Ammonium persulfate	200 g.s	Biocompatible (in vitro)	Tissue adhesive	[58]
Elastin-derived polypeptides	DA/carbodiimide chemistry	Oxidative/NaIO_4_	37 kPa	Biocompatible (in vivo) Low inflammatory response Suitable degradation profile (>10 weeks)	Wound healing and wound dressing	[59]

DA: dopamine; NHS: N-Hydroxysuccinimide; L-DOPA: 3,4-dihydroxyphenyl-L-alanine; UV: ultraviolet; NaIO_4_: sodium periodate; MgSO_4_: magnesium sulphate; CaSO_4_: calcium sulphate; p-NPC: p-nitrophenyl chloroformate; DETA: diethylenetriamine; TETA: triethylenetetramine; RGD: arginine-glycine-aspartate; HATU: 1-[Bis(dimethylamino)methylene]-1H-1,2,3-triazolo[4,5-b]pyridinium 3-oxide hexafluorophosphate; N.A.: not applicable.

##### Polyethylene Glycol (PEG)

PEG is a highly hydrophilic polyether compound, with wide applications in the pharmaceutical and biomedical fields. Also known as polyethylene oxide (PEO, depending on the Mw), PEG is obtained from the polymerisation of ethylene oxide, with a wide range of Mw (0.3–10,000 KDa) commercially available. Adhesive PEG-based biomaterials have been described for clinical applications, especially as tissue adhesives/sealants.

Li et al. [50] reported the development of a novel hybrid bioadhesive system, combining photo-crosslinking capable urethane methacrylated dextran (Dex-U) and catechol-functionalised PEG (PEG-DOPA), obtained via Michael reaction between acrylate bonds (introduced in PEG) and amines of DA (DS = 30%). Hydrogels were formed upon the UV irradiation of Dex-U and PEG-DOPA blend solutions, with quick polymerisation kinetics (under 2 min) proportional to the content of Dex-U in the blend. Lap shear tests using gelatine as a tissue mimetic revealed increased adhesion strength for constructs with higher PEG-DOPA content (4.0 MPa vs. 2.7 MPa for Dex-U hydrogels), with similar observations found in burst pressures tests (highest values for both tests were obtained at Dex-U/PEG-DOPA weight ratio of 3/2). Moreover, cytotoxicity assays in L-929 murine fibroblasts using hydrogel extracts revealed no significant toxicity of Dex-U/PEG-DOPA constructs.

To investigate the effect of pH on the gelation and adhesivity of catechol-containing PEG hydrogels, Cencer et al. [47] prepared DA-modified PEG (PEG-DA) hydrogels by the conjugation of DA with NHS-modified four-armed PEG via amine coupling chemistry (DS = 94 ± 6%). The curing rate, mechanical and adhesive properties of oxidatively NaIO_4_-mediated crosslinked hydrogels were investigated by the authors, with findings indicating that the pH plays an important role in the oxidative intermolecular crosslinking of catechol-containing adhesives. Indeed, under mild acidic conditions (pH = 5.7), hydrogels crosslinked at a slower rate (~6 min) compared to moderate alkaline conditions (pH = 8.0, ~20 s), which was associated with worse mechanical performance in compression tests, most likely due to less extensive DA oxidation. Optimal mechanical performance was obtained for reactions at near-physiological pH (7.4), with adhesives formulated under these conditions also displaying increased adhesive strength in tests performed with pericardium substrates. Importantly, by showing that adhesive strength and adhesion values for fully cured hydrogels (7.8 ± 1.7 kPa and 15 ± 2.0 J/m^2^, respectively) were superior to those obtained for constructs allowed to partially cure for 1 h (3.3 ± 0.52 kPa and 4.8 ± 1.0 J/m^2^, respectively), the authors demonstrated that the adhesive properties of the PEG-DA constructs were related to the oxidative state of catechol.

Catron et al. [48] described a simple approach for improvements of the mucoadhesive properties of four-armed PEG, via modifications of each terminal with L-DOPA using standard carbodiimide coupling chemistry (DS = 6%). Using a variety of surface analytical techniques, including single molecule atomic force microscopy (AFM) and X-ray photoelectron spectroscopy (XPS), the authors demonstrated a surprisingly strong interaction between DOPA and titanium-oxide-supported mucin (371 pN) across a range of acidic and alkaline pH values (4.5 to 8.5), which was significantly higher than that found for the control non-modified PEG or other mucoadhesive substrates (including PAA, Chi and pectin-based GZ-955), thus confirming DOPA-dependent mucoadhesive strength. 

In innovative studies from the same research group, aimed at imparting controllable adhesive properties to PEG for the development of improved liquid bioadhesives, Burke et al. [49] designed a smart strategy for the sequestration of DOPA-modified PEG (synthesised via standard carbodiimide coupling chemistry) and oxidant NaIO_4_ inside lipid vesicles, for controlled release and polymer crosslinking at physiological temperature. Thermal induction of hydrogel crosslinking (triggered by NaIO_4_ release at lipid phase transition temperature) was monitored via colorimetric assay (oxidation of DOPA measured at 320 nm), with rheological measurements revealing a rapid increase in G’ (i.e., gelation) at 37 °C and stabilisation over time, to a final elastic profile. Importantly, tissue adhesive properties of the hydrogels, assessed in porcine skin using lap shear tests, revealed higher adhesive strength for DOPA-modified PEG hydrogels (35 kPa) compared to unmodified PEG constructs or commercially available fibrin glue (6.9 kPa), thus providing further evidence of the beneficial effects of DOPA modification in the adhesive properties of PEG.

##### Cyanoacrylate Derivatives

Cyanoacrylates are a family of strong synthetic adhesives derived from ethyl cyanoacrylate and related esters, which undergo rapid polymerisation in the presence of water. With multiple industrial and medical applications, cyanoacrylates are nevertheless limited by toxicity towards cells and tissues.

In an attempt to improve the biocompatibility and adhesion strength of cyanoacrylate bio-glues, Lim et al. [51] combined pre-polymerised allyl 2-cyanoacrylate (PAC) with DA or L-DOPA via simple mixing at different ratios. Initial experiments focused on determining the ideal reaction co-initiator, with rheological measurements revealing decreasing setting times with increasing concentrations of L-DOPA or DA. The polymerisation reaction with L-DOPA was significantly slower than that with DA (curing times < 1 h); therefore, subsequent experiments were performed with the DA substituent. Various properties of polymer mixtures were assessed, namely, adhesion/bond strength, crystallisation, and cytotoxicity. In this regard, lower toxicity and higher adhesive strength (0.71 MPa) were found for a polymer mix containing 5 mg of DA, compared to octyl 2-cyanoacrylate (0.2 MPa), thus supporting the role of DA in improving the biological properties of cyanoacrylate glues.

Combinations of cyanoacrylate derivatives with PEO-PPO-PEO block copolymers were reported in two separate studies.

In a study of novel biomimetic adhesives for applications in bone fixation procedures, Jo et al. [52] designed mussel-inspired constructs composed of PEO-PPO-PEO functionalised with adhesive catechol. For this purpose, the authors prepared DA methacrylamide (DMA) by reacting DA with methacrylate anhydride (63% yield), which was further reacted with 2-methoxyethyl acrylate (MEA) and ethyl 2-cyanoacrylate (ECA) to generate copolymers of varied chain length, with the most suitable adhesive properties obtained for a blend containing 25% DMA, 25% MEA and 50% ECA. Mixtures of DMA-MEA-ECA with PEO-PPO-PEO formed complex coacervate structures (i.e., a type of lyophilic colloid) upon mixing with Ca^2+^ and Mg^2+^, with bone adhesion testing revealing the highest values in stress at break for biomimetic adhesives prepared in a wet state with Ca^2+^ and Mg^2+^ (165 KPa). Anion–cation interactions between catechol groups Ca^2+^/Mg^2+^ were suggested to have a stronger effect on the adhesion strength and modulus.

Similarly, Kang et al. [53] described the synthesis of injectable and temperature-sensitive hydrogels for use as tissue adhesives by crosslinking DA end-capped Pluronic (Plu-DP) and CCDP-q-PDB (2-chloro-3′,4′-dihydroxyacetophenone-quaternized poly((dimethyl aminoethyl methacrylate)-co-(tbutylmethacrylate))). The mixture of Plu-DP and CCDP-q-PDB (14:8 wt.% ratio) was a viscous solution at room temperature (15 °C), whereas rapid in situ crosslinking occurred upon exposure to body temperature (37 °C) and physiological conditions (< 10 s). The gel strength of the Plu-DP/CCDP-q-PDB hydrogels remained unchanged during the reversible sol–gel–sol transitions, while assessment of erosion rates revealed hydrogels with excellent stability under physiological conditions. Quantitative assessment of the tissue-adhesive properties of Plu-DP/CCDP-q-PDB hydrogels, using a UTM, revealed an increase of 8 KPa in the adhesive force of DA-modified hydrogels (compared to unmodified constructs), thus confirming the impact of catechol in the adhesive strength of the hydrogels. In vitro cytotoxicity studies showed that the polymer or its biodegraded form did not negatively affect the viability of MDCK epithelial kidney cells across a wide range of concentrations (0.1–1 mg/mL), thus confirming the biocompatibility of Plu-DP/CCDP-q-PDB hydrogels.

##### Recombinant Proteins

Recombinant proteins, obtained by the manipulation of naturally occurring proteins via bioengineering/DNA recombination, have also been tested for the preparation of bioadhesive hydrogels.

Aiming to develop protein-based adhesives for wound closure, Desai et al. [59] designed a hydrogel combining flexible and thermo-responsive elastin-derived polypeptides (ELPs) with adhesive catechol moieties. For this purpose, the authors modified recombinant ELPs (containing repetitive units of the pentapeptide VPGXG), as well as ELPs with RGD adhesion motifs in NH_2_/COOH terminals (ELP-RGD), via reactions with DA using EDC/NHS coupling chemistry (maximum DS of 4.1%). In addition to NMR spectroscopy, functionalisation was confirmed by the detection of a significant decrease in the transition temperature of modified proteins (Cat-ELPs) (16–28 °C) compared to the native counterparts (>90 °C). Stable swelling under water at physiological temperature (85–89% water content) was found for different oxidatively crosslinked hydrogels, with rheology measurements revealing rapid hydrogel crosslinking (within 10 min of NaIO_4_ mixing), and G’/G’’ increasing during curing (~1 h). Importantly, higher G’ values were detected for constructs with higher catechol content (without alterations in G’’), thus suggesting that interactions between catechol groups contribute to higher crosslinking density and hydrogel stiffness. Cell adhesion on the surface of constructs was tested with NIH3T3 mouse fibroblasts, with moderate cell spreading found for Cat-ELPs (proportional to catechol content), which was significantly improved in the RGD-containing composites. Adhesiveness of Cat-ELPs to biological tissues was evaluated in porcine skin, with tensile and shear strength testing revealing stronger adhesivity for Cat-ELPs with higher catechol content (39 and 37 kPa, respectively). Finally, in vivo biocompatibility and biodegradation of subcutaneously implanted Cat-ELPs, Cat-ELP-RGD or sham controls in mice, followed by monitoring over a period of 10 weeks, revealed reduced immune cell infiltration, with hydrogels completely degrading over the testing period.

Kim et al. [54] were able to convert tyrosine residues present in a recombinant fp-1 mussel adhesive protein (MAP) into DA by the action of mushroom Tyr, with gelation achievable via oxidation-induced quinone-mediated covalent crosslinking or Fe^3+^-mediated noncovalent crosslinking. In this regard, ionic gelation (and resulting hydrogels) was found to be superior to that induced by the oxidation of catechol groups (and resulting hydrogels), including (i) quicker coordinative interaction, and (ii) more favourable deformability and self-healing viscoelastic behaviour of hydrogels. Interestingly, whereas superior bulk adhesion strength towards “wet” porcine skin was found for oxidatively-crosslinked hydrogels (~200 kPa vs. ~130 kPa) in lap shear tests, further measurements demonstrated that Fe^3+^-mediated crosslinked hydrogels better retained their initial adhesive strength (over six cycles), whereas oxidatively crosslinked hydrogels lost adhesive capacity after four cycles. Overall, this manuscript has highlighted the potential of MAP hydrogels for future use as tissue adhesives.

##### Other Synthetic Polymers

Modification of synthetic biomaterials with adhesive moieties often requires multiple reaction steps for surface functionalisation, which impacts process scalability and downstream applications. Aiming at overcoming this limitation, Spaans et al. [57] developed a supramolecular approach based on ureido-pyrimidinone (UPy)-modified polymers combined with catechol chemistry to improve cell adhesion onto supramolecular biomaterials. This was achieved by synthesising UPy-DA via the amide coupling of DA to UPy-COOH (87% yield), followed by modular incorporation of UPy-DA onto the non-adhesive UPy-modified Priplast (an amorphous polyester polyol); a more adhesive UPy-PCL construct was used as a control. Dropcast films with fibrous morphology and rough surface were produced by the mixing of different UPy-polymers with UPy-DOPA, with Arnow’s tests (for the detection of catecholates) confirming the presence of catechol moieties. Importantly, films of UPy-polymer containing UPy-catechol (at 5 and 10 mol%) were found to support the adhesion and proliferation of cardiomyocyte progenitor cells (CMPCs), enhance the expression of CMPC markers, and preserve ECM production, as result of the modification with catechol. Indeed, no improvement in CMPC adhesion was detected in experiments using UPy-Priplast or UPy-PCL combined with catechol-free UPy-MeO, thus highlighting the role of catechol in the improved cell adhesion of UPy-Priplast.

Similarly, Wei et al. [55] described the development of stretchable, bioactive and adhesive supramolecular hydrogels for biomedical applications, based on multivalent host–guest macro-crosslinkers (HGMCs). This strategy for the fabrication of hydrogels is built on a backbone polymer unit combined with self-assembling guest polymers and host monomers, with crosslinking driven by molecular recognition between the two entities. In this study, acrylamide (AAm) was used as a monomer for hydrogel preparation, thiourea (TU)-linked monoacrylated β-cyclodextrins (Ac-TU-βCD) as host monomers, and adamantane-functionalized HA (AD-HA) as guest polymer. The authors demonstrated that the gelation, dynamic modulus and physical properties of hydrogels could be tuned by adjusting the degree of adamantane modification and the solid content of the guest polymer (AD-HA), or simply by adjusting the molar ratio between host and guest moieties. TU-HGMC constructs capable of stretching up to 17 times their original length and conformational recovery upon removal of the stimulus were confirmed by oscillatory frequency-sweep measurements. TU-HGMCs are biologically inert; therefore, bioactivation with a catechol-modified peptide (Cat-RGD) for enhanced adhesion and cell attachment, was achieved post-gelation via thiourea−catechol (TU-Cat) coupling chemistry. Indeed, functionalised TU-HGMC hydrogels supported excellent cell attachment and the spreading of hMSCs seeded on the surface of the constructs, compared to control HGMC gels, as well as enhanced (but reversible) catechol-mediated adhesion to porcine skin and liver.

Joshi et al. [58] reported the development of polyacrylic acid (PAA) and alginic acid (Alg) adhesive hydrogels for biomedical application. Unmodified PAA and Alg present interesting properties for the preparation of hydrogels, including ease of chemical modification, pH sensitivity, and capacity to retain water, which is nevertheless limited by poor adhesion. Taking advantage of the capacity of nucleobases and derivatives to form supramolecular structures through intermolecular hydrogen bonds, the authors fabricated double-modified (catechol and adenine) PAA (compound P2) in a two-step process, which involved the synthesis of an adenine-DA adduct followed by conjugation with PAA via carbodiimide (EDC/NHS) coupling chemistry (DS = 0.1%); adenine-modified PAA (no DA) was synthesised as a control (compound P1). Ammonium-persulphate-induced gelation resulted in transparent hydrogels, although P2 presented a lightly yellow coloration, most probably due to the oxidation of catechol. P2 hydrogels were found to have superior performance, compared to P1 and PAA hydrogels, regarding (i) swelling, (ii) elastic behaviour (G’ > G’’), and (iii) adhesiveness. P2 demonstrated excellent adhesivity to soft and hard surfaces, including metal, plastic, leaf, wood and porcine skin, without leaving any residues upon detachment, which was further confirmed by the quantitative analysis of hydrogel adhesion to glass, aluminium and porcine skin. Interestingly, the combination of P2 with Alg polymers (P4) further enhanced the mechanical properties (G’ P2P4 > G’ P2) and bioactivity of the hydrogels. Indeed, 2D and 3D cell culture experiments, using BHK-21 baby hamster kidney fibroblasts and A431 skin cancer cells, revealed increased cell density for cells incubated in P2P4 hydrogels for 24 h, compared to individual polymer components.

Guvendiren et al. [56] prepared a series of adhesive self-assembly-capable hydrogels via the functionalisation of polymethyl methacrylate–polymethacrylic acid–polymethyl methacrylate (PMMA–PMAA–PMMA) triblock copolymers with DA. Three different constructs with three different DA levels (0, 20 and 40 mol%) were generated by the incorporation of DA in the midblock of the copolymer, with in situ hydrogel formation achieved by vapor phase solvent exchange. The mechanical and adhesive properties of the resulting hydrogels were found to be affected by the pH of buffers covering the hydrogels, which affect the protonation of DA. Indeed, the authors demonstrated that, at pH 6 and pH 7.4, DA decreases the hydrogel swelling capacity and increases adhesive strength towards titanium oxide surfaces at sufficiently long contact times (~2 J/m^2^ for interfacial fracture energy), due to the hydrophobicity of fully protonated DA. Importantly, alkaline buffers (pH = 10) were found to increase hydrogel swelling and decrease adhesive performance, due to the accelerated oxidation of DA, thus confirming the important role of DA in the adhesive and swelling properties of the produced gels.

## 4. Discussion

Biomaterial adhesion in wet environments is significantly impacted by the presence of water molecules, with a widely recognised decrease in adhesion strength due to (i) the formation of a hydration layer in the biomaterial/tissue interface which limits the contact between molecules, (ii) water interference with the electrostatic and hydrophobic interactions between biomaterials and tissues, and (iii) swelling of the biomaterials leading to weakened adhesive forces [60].

Several strategies can be employed to minimise these adverse effects, including the optimisation of biomaterial surfaces to decrease hydrophilicity/wettability (and therefore decrease the number of water molecules bound to the surfaces) and the fine-tuning of biomaterial viscosity to increase the formation of interlocking structures with tissues, which are less prone to be affected by water. Indeed, high underwater adhesion has been reported for biomaterials capable of establishing strong interlocking structures with wet substrates. Suo et al. [61] described the preparation of slug-inspired tough adhesives (TAs), combining alginate and a bridging polymer in a two-layer structure, which displayed high adhesion strength mediated by strong interlocking interactions between adhesive and substrate, as well as energy dissipation through hysteresis. Specialised adhesive structures are also found in nature, especially in animals or microorganisms living in wet conditions or requiring movements in extreme plans (e.g., keratin foot-hairs lining the sole of gecko’s feet), which rely on micro-topography cues for enhanced adhesivity to surrounding surfaces [62]. To date, however, mussels constitute the largest source of inspiration for the development of adhesives with improved wet adhesive strength, as confirmed by the findings of this literature review. Indeed, catechol-conjugated, tissue-adhesive polymers with tuneable physical and mechanical properties have been described for a range of natural origin biomolecules, including (i) polysaccharides such as chitosan, hyaluronic acid, alginate, dextran, cellulose, xanthan gum, gellan gum, and (ii) proteins such as gelatine and silk fibroin, as well as synthetic polymers such as polyethylene glycol (PEG) and cyanoacrylate derivatives. The common theme of all articles reviewed herein centres around the desire to improve the adhesion of hydrogels prepared from such polymers to soft and hard tissues or surfaces for applications in tissue engineering and regenerative medicine. Overall, there is a sufficient weight of evidence that the chemical modification of biomaterials with dopamine (DA) or other catechol-containing substituents leads to a viable semi-synthetic approach for mimicking marine-inspired bioadhesion processes found in nature and provides substantial improvement over the properties of the parent polymer.

In addition to the impact of catechol modification on adhesivity, this review also harnesses additional information which imparts further incentives for modifications of biomaterials with catechol moieties, namely:

(i) The majority of synthetic methods for the grafting of DA or other catechol-containing substituents to polysaccharide polymer systems involves relatively straight-forward carbodiimide (amidation) chemistry. GG shares a free carboxyl group with most of the above-mentioned polysaccharides in each tetrasaccharide repeat unit for coupling with DA;

(ii) Catechol-modified polymers provide remarkable versatility in terms of cross-linking and hydrogel-forming capability. Although the parent polymers are normally limited in this aspect, the covalently conjugated catechol group permits cross-linking via ionic, oxidative, enzymatic and pH-controlled mechanisms, either alone or in combination;

(iii) Substitution by DA or other catechol-containing substituents does not generally cause negative impacts on the gelling kinetics and mechanical properties of hydrogels compared to the parent polymer. Indeed, in several studies, improved gelation and mechanical properties were associated with biomaterial functionalisation with catechol substituents;

(iv) Catechol functionalisation does not generally lead to any significant loss of biocompatibility (e.g., cytotoxicity) compared to the parent polymer systems;

(v) Substitution by DA or other catechol moieties does not generally lead to any significant loss of cell viability or proliferation of cells encapsulated in hydrogels compared to the parent polymer. Indeed, in a large number of studies, enhanced viability/proliferation was associated with modifications of the biomaterial with catechol functional groups.

Based on the above listed findings, it was anticipated that the same observations would apply to GG once substituted by DA. Indeed, in our own published study [40] (which is included in the list of selected manuscripts), a mussel-inspired DA-modified GG hydrogel was designed for minimally invasive delivery in cartilage repair procedures. Aiming at improving on GG limitations, including low aqueous solubility and poor adhesivity, the parent polymer was initially purified to a monovalent sodium salt form (GGp) and further functionalised with DA upon the activation of carboxylic groups with 4-(4,6-dimethoxy-1,3,5-triazine-2-yl)-4-methylmorpholinium chloride (DTMMCI) (DS = 4.7%). DA-modified GGp (STM-148B) solutions (1% *w*/*V* in water) displayed a favourable shear-thinning profile (i.e., decreasing in solution viscosity over the shear rate 0.01–1000 s^−1^) and a dynamic mono- and divalent cation-mediated crosslinking process, with a moderate elastic profile in the first 5 min after mixing with the crosslinker (PBS Ca^2+^/Mg^2+^), followed by a significant G’ increase after hydrogel submersion in NaCl (0.9% (*w*/*V*)), reaching an equilibrium after 10 min. Similarly, G’ > G’’ was found for STM-148B hydrogels for all tested frequencies. Moreover, STM-148B hydrogels displayed suitable water retention and swelling capacity (140% of initial volume), crosslinker-dependent porosity, and improved adhesiveness to cartilage defects created in porcine knee, compared to GGp or photo-crosslinked methacrylated GG. Extensive in vitro studies were carried out to characterise the biological performance of STM-148B hydrogels, with cytotoxicity assays revealing no significant toxicity of hydrogel extracts towards L-929 mouse fibroblasts, primary ovine knee chondrocytes, human nasal chondrocytes (hNCs), and human adipose-derived MSCs (hASCs). Moreover, STM-148B hydrogels effectively maintained the viability of encapsulated hNCs or hASCs for up to 21 days in culture, while supporting the production of ECM components (including collagen II, aggrecan and proteoglycans) by encapsulated hASCs upon stimulation with chondrogenic differentiation factors. Subsequent in vivo proof-of-concept studies carried out in an ovine model of disease confirmed the safety and performance of the biomaterial.

Importantly, the versatility and breadth of applications for GGp is not limited to cartilage repair. In addition to DA modification (for enhanced adhesivity), our group is exploring a biomaterial-based formulation development platform for the de novo synthesis of customised chemical entities, towards applications in cell encapsulation/delivery and tissue regeneration (including 3D printing).

## 5. Conclusions and Future Perspectives

**Figure 7:**
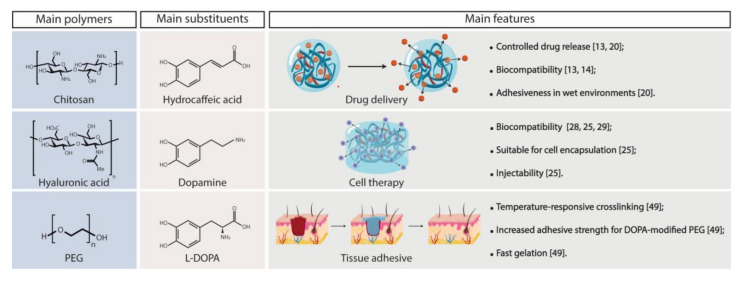
Chemical structures of polymers and substituents more frequently cited in this systematic review, as well as the main findings reported those studies.

Overall, the data presented herein support the rationale for the functionalisation of naturally occurring or synthetic polymers with catechol moieties, towards the development of optimised biomaterials with improved adhesive performance in dry and (especially) wet “physiological-like” conditions. Such improvements have contributed to the development of a novel generation of biomaterials, capable of surpassing commercially available tissue adhesives and wound dressings in their haemostatic potential and wound healing properties. Moreover, improved adhesion of these biomaterials has shown to be a valuable asset for controlled drug delivery systems and cell therapies that rely on the interaction with wet mucosa (Figure 7). Inspired by marine organisms, these innovative biomaterials show great promise in reaching clinical applications by supporting improved formulation strategies of medical devices and new advanced therapy medicinal products (ATMPs) for treating life-threatening conditions such as acute bleeding or cancer, among others.

## Figures and Tables

**Table 1:** Appraisal criteria for assessment of manuscripts towards inclusion/exclusion from the final reference list.

Data Contribution Criteria	Description	Grading System	
**Data Source Type**	Was the design of the biomaterial appropriate?	T1 T2	Yes No
Functionality outcome	Does it report improvements in adhesion of the biomaterial as result of the modification with dopamine or a catechol-containing substituent?	F1 F2	Yes No
Outcome measures	Does the reported improvement in adhesion reflect the intended performance of the biomaterial?	O1 O2	Yes No
Comparative significance	Has a comparative analysis of the data been provided with the parent polymer or other control group and is it appropriate to determine a clear cause–effect relationship for the dopamine/catechol substituent?	S1 S2	Yes No

**Table 2:** Data relevance for manuscript inclusion/exclusion in the final reference list.

Relevance Level	Criteria
High relevance to design assessment	Total score of 4
Relevant to design assessment	Total score of 5
Low relevance to design assessment	Total score of 6 or 7
Negligible relevance to design assessment	Total score of 8

**Table 3:** Studies involving naturally sourced polymers, selected for inclusion.

Polymer	Substituent/ Coupling Chemistry	Crosslinking Mechanism/Catalyst	Adhesive Strength	Reported Features/Findings	Intended Application	Ref.
Chitosan	HCA/carbodiimide chemistry	Chemical/genipin	Up to 6 h (compared to 1.5 h for non-modified polymer)	Slow degradation Controlled drug release Biocompatible (in vitro/in vivo) Low inflammatory response	Drug delivery	[13]
	HCA/carbodiimide chemistry	N.A.	4-fold increase over non-modified polymer	Biocompatible (in vitro)	Drug delivery	[14]
	HCA/carbodiimide chemistry	Ionic/hematin	33.6 ± 5.9 kPa	Enhanced solubility and activity Biocompatible (in vitro)	Tissue adhesive	[15]
	L-DOPA/carbodiimide chemistry	Chemical (Michael-type addition)	15.0 ± 3.5 kPa	Thermosensitive Injectable Stable (in vitro/in vivo)	Drug delivery and tissue engineering	[16]
	DHCA/carbodiimide chemistry	Oxidative/NaIO_4_	50 kPa	Biocompatible (in vitro) Fast in situ curing	Tissue adhesive	[17]
	HCA/Tyrosinase or carbodiimide chemistry	Oxidative/tyrosinase	64.8 ± 5.7 kPa	Biocompatible (in vitro) Improved healing performance (in vivo)	Wound dressing	[18]
	CA/carbodiimide chemistry	Oxidative/MnO_2_	3.35 kPa	Self-healable Shear thinning behaviour Controlled drug release Photo-mediated increase in local temperature Accelerated wound closure	Cancer therapy	[19]
	HCA/carbodiimide chemistry	N.A.	10.3 KPa to 20 KPa (porcine mucosa)	Dual release profile Adhesive in wet environments	Drug delivery	[20]
Chitosan and hyaluronic acid	DA/carbodiimide chemistry	Ionic	>60 % of Cat-NPs remain attached to buccal mucosa after rinsing with artificial saliva	Biocompatible (in vitro) High drug-loading efficiency	Drug delivery	[21]
Chitosan and ε-polylysine	DOHA/carbodiimide chemistry	Chemical (Michael addition)	0.185 N	Nerve regeneration (in vivo) Biocompatible (in vitro/in vivo) Low immunogenicity	Tissue regeneration, adhesive	[22]
Chitosan and gamma-polyglutamic acid	DPA and DA/carbodiimide chemistry	Photo-mediated (UV light)	25 kPa (porcine skin) and 145 kPa (arthrodial cartilage)	Suitable degradation profile (>1 week) Biocompatible (in vitro)	Adhesive	[23]
Hyaluronic acid	DA/carbodiimide chemistry	Polyelectrolyte interaction	2.0 ± 0.2 MPa	Biocompatible (in vitro) Anti-inflammatory Resemblance to native ECM	Wound dressing	[24]
	DA/carbodiimide chemistry	Oxidative/NaIO_4_	1.356 ± 0.084 kPa	Biocompatible (in vitro) Tuneable gelation Suitable for cell encapsulation Injectable	Cell therapy	[25]
	DA/aldehyde-amine Schiff’s reaction	Oxidative/NaIO_4_ and NaOH	90.0 ± 6.7 kPa	Biocompatible (in vitro) Fast gelation (60 s) Tuneable degradation	Tissue adhesive	[26]
	DA/carbodiimide chemistry	In situ free radical polymerisation	49.6 kPa	High toughness Ultra-stretchable Self-healable	Strain sensor	[27]
	DA/carbodiimide chemistry	Chemical/hydrazone	Greater adhesion strength for modified hydrogels	Biocompatible (in vitro) Stable elastic hydrogels	Cell therapy	[28]
	DA/carbodiimide/HOBt coupling chemistry	Oxidative/NaIO_4_	Greater adhesion strength for modified hydrogels	Biocompatible (in vitro) Sustained drug release	Cell therapy and drug delivery	[29]
Silk fibroin	DA/COMU coupling mechanism	Oxidative/NaIO_4_	130 kPa	Biocompatible (in vitro)	Tissue adhesive	[30]
	L-DOPA, enzymatic reaction	N.A.	0.97 MPa	N.A.	Tissue adhesive	[31]
	Tyramine/COMU or CDI coupling mechanism	Oxidative/Tyrosinase	82.5 kPa (carboxylated silk fibroin) and 85 kPa (silk fibroin)	Biocompatible (in vitro) Fast gelation kinetics	Wound healing	[32]
	DA/carbodiimide chemistry	Chemical (pegylation)	217 kPa	Slow degradation rate	Tissue adhesive	[33]
Alginate	DA/carbodiimide chemistry	Ionic/GDL	Up to 7 h post implantation within pig abdomen	Biocompatible (in vitro) Wound healing properties Appropriate degradation kinetics	Tissue adhesive	[34]
	DA/carbodiimide chemistry	Ionic/calcium carbonate	0.24 N/m	Decreased elastic modulus with substitution Time- and pH-dependent adhesion	Tissue adhesives or wound dressing	[35]
Gelatine	DA/carbodiimide chemistry	Ionic/Fe^3+^	>2 N/m^2^	Suitable degradation profile (over 23 days)	Wound dressing	[36]
	DA/carbodiimide chemistry	Ionic/Fe^3+^	10–16 kPa (porcine myocardium/skin)	Conductive Regenerative properties Able to withstand twisting, bending and soaking in water	Tissue adhesive and wound healing	[37]
Gelatine methacrylate	DA/carbodiimide chemistry	Photo-mediated (UV light)	6.8 ± 0.55 kPa	Antibacterial Anti-inflammatory Successful sealing of bleeding wounds	Wound healing	[38]
	DA/carbodiimide chemistry	Oxidative/NaIO_4_	2 kPa	Rapid gelation 3D bioprintable Suitable for cell encapsulation Biocompatible (in vitro) Suitable degradation profile (in vivo) Low inflammatory response	Bioink	[39]
Gellam Gum	DA/carbodiimide chemistry	Ionic/PBS Ca^2+^/Mg^2+^	Up to 12 h	Xeno-free Injectable Suitable mechanical properties Biocompatible (in vitro) Suitable for cell encapsulation	Tissue engineering and cell therapy	[40]
Xanthan gum	DA/carbodiimide chemistry	N.A.	>25 kPa	Self-healable Fast degradation (in vitro) Anti-inflammatory	Wound healing	[41]
Chondroitin sulphate	DA/carbodiimide chemistry	Oxidative/NaIO_4_ and NaOH)	>2.5 N	Biocompatible (in vitro) Low immunogenicity	Tissue engineering and cell therapy	[42]
Dextran aldehyde and PEG amine	L-DOPA/dynamic imine (amine-aldehyde) chemistry	N.A.	Modification with L-DOPA led to less variation of burst pressure	Biocompatible (in vitro) Decrease in hydrogel strength with substitution	Wound dressing	[43]
Carboxymethyl cellulose	DA/carbodiimide chemistry	Chemical (Schiff’s base reaction)	86 kPa	Biocompatible (in vitro) Suitable adhesive profile (up to 2 weeks)	Tissue adhesive	[44]
	DA/carbodiimide chemistry	Enzymatic/HRP	28.5 kPa	Injectable Biocompatible (in vitro) Improved elasticity Suitable degradation profile (>1 week)	Wound dressing, wound healing and tissue engineering	[45]
Lignin	Quinone/redox reaction	Oxidative/Ammonium persulfate	27.5 kPa	Long-lasting adhesiveness Stretchable Quick recovery after deformation Anti-bacterial	Wound healing	[46]

HCA: hydrocaffeic acid; L-DOPA: 3,4-dihydroxyphenyl-L-alanine; DHCA: dihydrocaffeic acid; DA: dopamine; DPA: 3,4-dihydroxyphenyl propionic acid; DOHA: dihydroxybenzenepropionic acid; CA: caffeic acid; NaIO_4_: sodium periodate; MnO_2_: manganese dioxide; COMU: 1-Cyano-2-ethoxy-2-oxoethylidenaminooxy)dimethylamino-morpholino-carbenium hexafluorophosphate; CDI: 1,1′-carbonyldiimidazole; GDL: glucono-delta-lactone; NaOH: sodium hydroxide; HRP: horseradish peroxidase; UV: ultraviolet; N.A.: not applicable.

## Data Availability

The data presented in this study are available on request from the corresponding author.

## Supplementary Materials

The following is available online at https://www.mdpi.com/article/10.3390/polym13193317/s1, Table S1: Database search results up to 02 March 2021.
