# Supplementary material for: Mussel-Inspired Catechol Functionalisation as a Strategy to Enhance Biomaterial Adhesion: A Systematic Review
Source: Polymers (Basel). 2021 Sep 28;13(19):3317. doi: 10.3390/polym13193317 (PMC8513061; doi:10.3390/polym13193317)
Supplement: Supplementary file 1 [file polymers-13-03317-s001.zip › polymers-1383362-supplementary.pdf]

Table S1. - Database search results up to 02 March 2021.

Search period	Database	Number of results	Search Strategy
**Initial** (Database inception to 30 March 2019)	**Pubmed®**	272	*((((dopa*[Title/Abstract] OR catechol[Title/Abstract])) AND (biomaterial OR biopolymer OR scaffold OR matrix OR hydrogel OR device)) AND (modified OR conjugated OR attached OR functionalized OR covalent)) AND (biomimetic OR mussel OR bioinspired OR adhes*)*
	**Embase®**	218	*((((dopa*[Title/Abstract] OR catechol[Title/Abstract])) AND (biomaterial OR biopolymer OR scaffold OR matrix OR hydrogel OR device)) AND (modified OR conjugated OR attached OR functionalized OR covalent)) AND (biomimetic OR mussel OR bioinspired OR adhes*)*
**Revision/Update** (01 April 2019 to 02 March 2021)	**Pubmed®**	126	*((((dopa*[Title/Abstract] OR catechol[Title/Abstract])) AND (biomaterial OR biopolymer OR scaffold OR matrix OR hydrogel OR device)) AND (modified OR conjugated OR attached OR functionalized OR covalent)) AND (biomimetic OR mussel OR bioinspired OR adhes*)*
	**Embase®**	82	*((((dopa*[Title/Abstract] OR catechol[Title/Abstract])) AND (biomaterial OR biopolymer OR scaffold OR matrix OR hydrogel OR device)) AND (modified OR conjugated OR attached OR functionalized OR covalent)) AND (biomimetic OR mussel OR bioinspired OR adhes*)*
**Overall** (Database inception to 02 March 2021)	**Pubmed®**	**398**	*((((dopa*[Title/Abstract] OR catechol[Title/Abstract])) AND (biomaterial OR biopolymer OR scaffold OR matrix OR hydrogel OR device)) AND (modified OR conjugated OR attached OR functionalized OR covalent)) AND (biomimetic OR mussel OR bioinspired OR adhes*)*
	**Embase®**	**300**	*((((dopa*[Title/Abstract] OR catechol[Title/Abstract])) AND (biomaterial OR biopolymer OR scaffold OR matrix OR hydrogel OR device)) AND (modified OR conjugated OR attached OR functionalized OR covalent)) AND (biomimetic OR mussel OR bioinspired OR adhes*)*
